# Responding to health needs of women, children and adolescents within Syria during conflict: intervention coverage, challenges and adaptations

**DOI:** 10.1186/s13031-020-00263-3

**Published:** 2020-05-29

**Authors:** Chaza Akik, Aline Semaan, Linda Shaker-Berbari, Zeina Jamaluddine, Ghada E. Saad, Katherine Lopes, Joanne Constantin, Abdulkarim Ekzayez, Neha S. Singh, Karl Blanchet, Jocelyn DeJong, Hala Ghattas

**Affiliations:** 1 Center for Research on Population and Health, Faculty of Health Sciences, American University of Beirut, Beirut, Lebanon; 2 Epidemiology and Population Health Department, Faculty of Health Sciences, American University of Beirut, Beirut, Lebanon; 3 King’s College, London, UK; 4 Health in Humanitarian Crises Centre, London School of Hygiene and Tropical Medicine, London, UK

**Keywords:** Reproductive health, Maternal health, Child health, Nutrition, Health interventions, Coverage, Syria, Armed conflict, Humanitarian crises

## Abstract

**Background:**

Women and children suffer disproportionately in armed-conflicts. Since 2011, the protracted Syrian crisis has fragmented the pre-existing healthcare system. Despite the massive health needs of women and children, the delivery of key reproductive, maternal, newborn, child and adolescent health and nutrition (RMNCAH&N) interventions, and its underlying factors are not well-understood in Syria. Our objective was to document intervention coverage indicators and their implementation challenges inside Syria during conflict.

**Methods:**

We conducted 1) a desk review to extract RMNCAH&N intervention coverage indicators inside Syria during the conflict; and 2) qualitative interviews with decision makers and health program implementers to explore reasons behind provision/non-provision of RMNCAH&N interventions, and the rationale informing decisions, priorities, collaborations and implementation. We attempt to validate findings by triangulating data from both sources.

**Results:**

Key findings showed that humanitarian organisations operating in Syria adopted a complex multi-hub structure, and some resorted to remote management to improve accessibility to certain geographic areas. The emergency response prioritised trauma care and infectious disease control. Yet, with time, humanitarian organisations successfully advocated for prioritising maternal and child health and nutrition interventions given evident needs. The volatile security context had implications on populations’ healthcare seeking behaviors, such as women reportedly preferring home births, or requesting Caesarean-sections to reduce insecurity risks. Additional findings were glaring data gaps and geographic variations in the availability of data on RMNCAH&N indicators. Adaptations of the humanitarian response included task-shifting to overcome shortage in skilled healthcare workers following their exodus, outreach activities to enhance access to RMNCAH&N services, and operating in ‘underground’ facilities to avoid risk of attacks.

**Conclusion:**

The case of Syria provides a unique perspective on creative ways of managing the humanitarian response and delivering RMNCAH&N interventions, mainly in the multi-hub structure and use of remote management, despite encountered challenges. The scarcity of RMNCAH&N data is a tremendous challenge for both researchers and implementing agencies, as it limits accountability and monitoring, thus hindering the evaluation of delivered interventions.

## Background

Armed conflict and violence have grave direct and indirect health implications for civilians, the majority of whom are women and children [1, 2]. The collapse of public services, health systems and social networks places a disproportionate burden on the health of women and children [1, 2], which can be further exacerbated when they are forcibly displaced from their homes and communities.

The Syrian conflict started in March 2011 when a popular uprising in the South of the country was met by a security response that resulted in further unrest and escalation within the country [3]. The ensuing deadly war led to the division of the Syrian territory among conflicting political factions, and the fragmentation of the country’s governance between the Government of Syria and opposition groups [4, 5]. The evolution of the conflict has been marked by constant shifts in political boundaries as a result of the volatile security context [6].

Prior to the crisis, Syria was classified as a middle-income country, with a robust national health system led by the public sector, with a growing private sector [7] and little reliance on civil society organisations [8]. National commitment contributed to nearly 30 years of progress in health indicators; with Syria achieving 85 and 68% of its Millennium Development Goals (MDGs) 4 and 5 targets^1^ respectively by 2008 (data on pre-conflict indicators are displayed in Additional File 1) [9, 10]. Since 2011, the protracted crisis, political destabilization, targeting and destruction of healthcare facilities, attacks on health workers, and an exodus of well-trained health professionals have led to the partial collapse and fragmentation of the healthcare system [11–13]. The fracturing of the Syrian health system has had detrimental effects on healthcare provision, and considerable consequences on population health, including an increased risk of infectious disease outbreaks [14] and challenges in accessing maternal and child health interventions [4].

With a population of around 21 million pre-conflict [15], Syria witnessed massive waves of population displacement. Currently, 6.2 million people are internally displaced within Syria [16], and there are 5.4 million registered Syrian refugees in neighboring countries [17]. Considering the dynamic shifts in conflict lines, the number of internally displaced persons (IDPs) increased as Syrians were forced to move multiple times before reaching a safe region [18]. The IDPs live in informal settlements^2^ [16], and their health needs are exacerbated by poor living conditions [5, 18], and their limited access to humanitarian aid [4]. In 2018, an estimated 11.3 million people in Syria were in need of humanitarian assistance in health, of whom 1.3 million were children under 5 years of age [16]. The number of women of reproductive age (15–49 years) living in Syria in 2017 was estimated to reach 3.3 million [19].

**Fig. 1:**
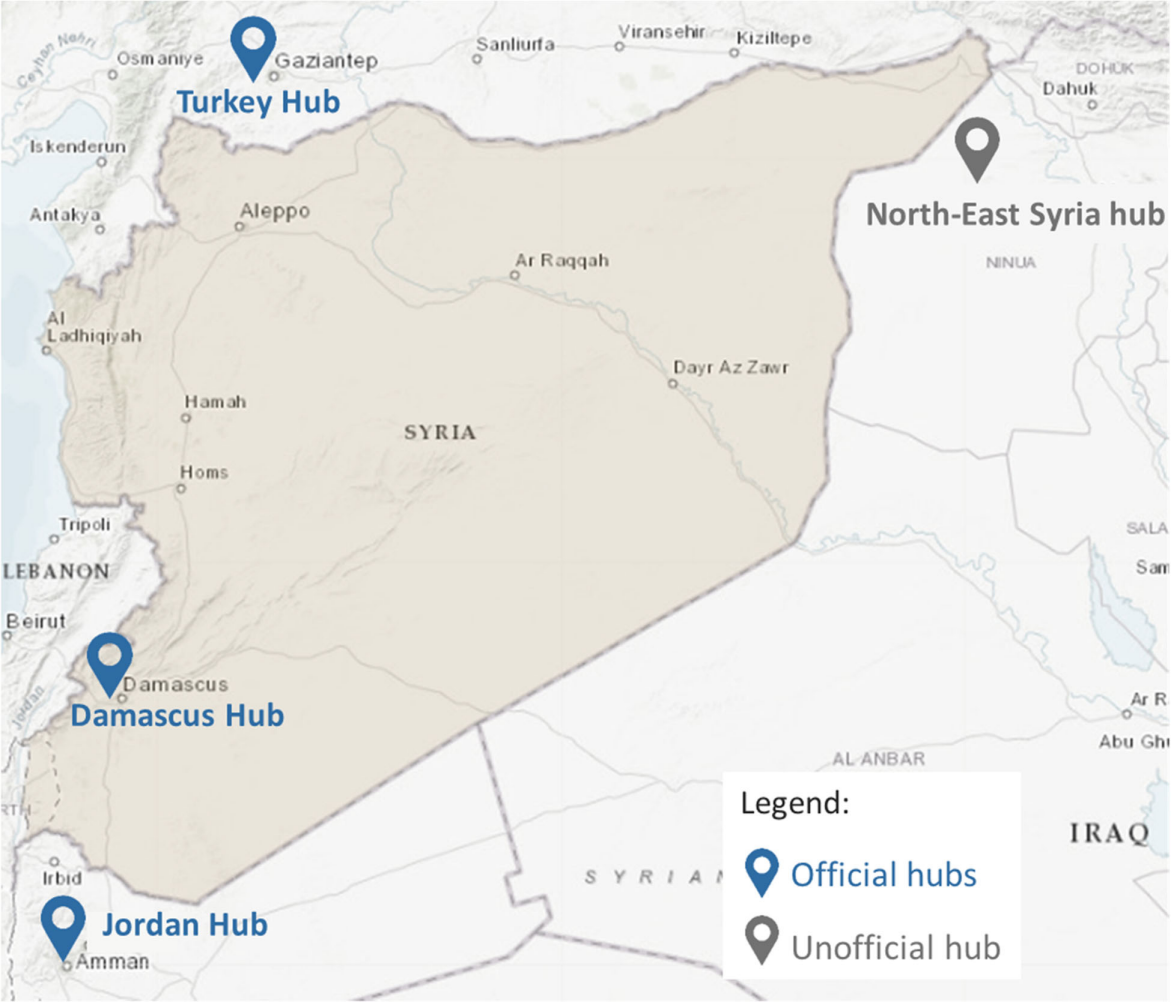
Hubs coordinating the humanitarian response in Syria

In light of the resulting humanitarian need, a large number of non-governmental organisations (NGOs) and United Nations (UN) agencies have established a humanitarian response in Syria [6]. Given the territorial and governmental fragmentation and insecurity, in addition to managing the response from inside Syria, humanitarian agencies have had to operate remotely from neighboring countries, following the United Nations Security Council (UNSC) resolution 2165^3^ adopted in 2014 [6, 20]. As a consequence, the humanitarian system adopted a complex structure which consists of three official coordination hubs: Damascus (Syria), Gaziantep (Turkey) and Amman (Jordan); and one unofficial hub serving the North-East of the country, all operating under the Whole of Syria framework [6, 21] (See Fig. 1). Within these structures, UN agencies collaborated with various governing bodies in areas under different control groups, such as the collaboration with the Syrian Ministry of Health in government-controlled areas, which was considered as a co-lead with WHO for the health cluster in the Damascus hub; or with the Health Directorates established by local health networks with a loose link to the interim ministry of health in opposition held areas [6, 22]. These collaborating bodies would contribute to the development of yearly Humanitarian Response Plans, based on regularly conducted Humanitarian Needs Overviews [16, 23].

Considering the specific needs and vulnerabilities of women and children during the Syrian conflict, it is unclear whether the remaining healthcare system supported by humanitarian interventions was able to deliver key reproductive, maternal, newborn, child and adolescent health and nutrition (RMNCAH&N) interventions [24]. These services included, but were not limited to, family planning, antenatal care (ANC), skilled birth attendance (e.g. EmOC, BEmOC, etc.), postnatal care (PNC) and newborn care, promotion of immediate and exclusive breastfeeding, and nutrition education and support [25–28]. In a recent review, we reported declines in coverage rates of certain key evidence-based interventions between January 2011 and December 2015 (e.g. measles immunization coverage among children 12–23 months decreased from 81.9% pre-conflict to 75% in 2013) [24]. The review also highlighted limitations in the data available on the health of populations remaining in Syria, as the majority of evidence focuses on Syrian refugees living in neighboring countries [24, 29, 30].

To better inform RMNCAH&N interventions and respond to women and children’s health needs in conflict, it is important to understand how the multiplicity of actors worked to deliver key interventions, what barriers were faced in the delivery of these interventions, and ways in which these barriers were overcome. As part of a multi-country study coordinated by the BRANCH^4^ Consortium and focused on RMNCAH&N in 10 conflict-affected countries (Afghanistan, Colombia, Democratic Republic of the Congo, Mali, Nigeria, Pakistan, Somalia, South Sudan, Syria, Yemen) [31] this research aimed to document the provision and coverage of RMNCAH&N interventions and explore the factors that influenced their implementation in Syria during the crisis. Ultimately, our purpose was to inform practices and policies to improve health service delivery and health outcomes for women, children and adolescents, and to derive potential lessons learned for other conflict settings.

## Methods

In order to achieve these objectives, this study adapted a common protocol developed for all case studies [31]. We used two main data sources: (1) an updated desk review of DeJong et al. [24] from which quantitative data on coverage indicators were extracted; and (2) qualitative key informant interviews with decision-makers and health program implementers. The approach was both exploratory and explanatory; each data source was used to generate new evidence and both sources were triangulated in an attempt to validate findings. For example, the results of the desk review were concurrently shared with the qualitative research team to refine probes for the topic guide and ask for clarifications from respondents about specific coverage indicators during the key informant interviews. Findings from the desk review were also iteratively used to complement the qualitative findings.

### Desk review

We conducted a desk review that built on the abovementioned previously conducted review [24], and used the same search strategy to systematically update numbers on RMNCAH&N coverage indicators for populations living in Syria. The objective of the desk review was to document coverage indicators during the 2011–2018 period of the conflict in comparison to pre-conflict baseline data when available. This was achieved by merging the findings of the first review (January 2011 to December 2015) with those of the updated search (January 2015 to March 2018), to which we added search terms for adolescents. We searched the same published and grey literature databases including but not limited to Medline, PubMed, Relief Web, WHO EMRO websites [24] (key words, search strategy and inclusion/exclusion criteria are detailed in Additional File 2).

We further explored the World Health Organisation (WHO) vaccine-preventable diseases monitoring system [32], from which we extracted annual immunization coverage estimates. We included all national estimates that present coverage rates published between 2010 and 2017. Additionally, we reviewed all published Health Resources and service Availability Mapping System (HeRAMS) reports, available only since 2014 [33], from which we extracted facility-level monthly Caesarean-section (C-section) rates in the included public facilities, between January 2014 and March 2018, with the exception of the year 2016 for which reports were not accessible.

We imported all retrieved documents into EndNote reference manager [34] and removed duplicate records. We included original research studies and reports that described relevant RMNCAH&N coverage indicators among the Syrian population residing in Syria, and documents reporting on interventions delivered and factors affecting their implementation during the Syrian conflict. Two researchers conducted title and abstract screening followed by full-text screening, independently. Disagreements were discussed among and resolved by the two reviewers, and with a third independent researcher if needed.

**Fig. 2:**
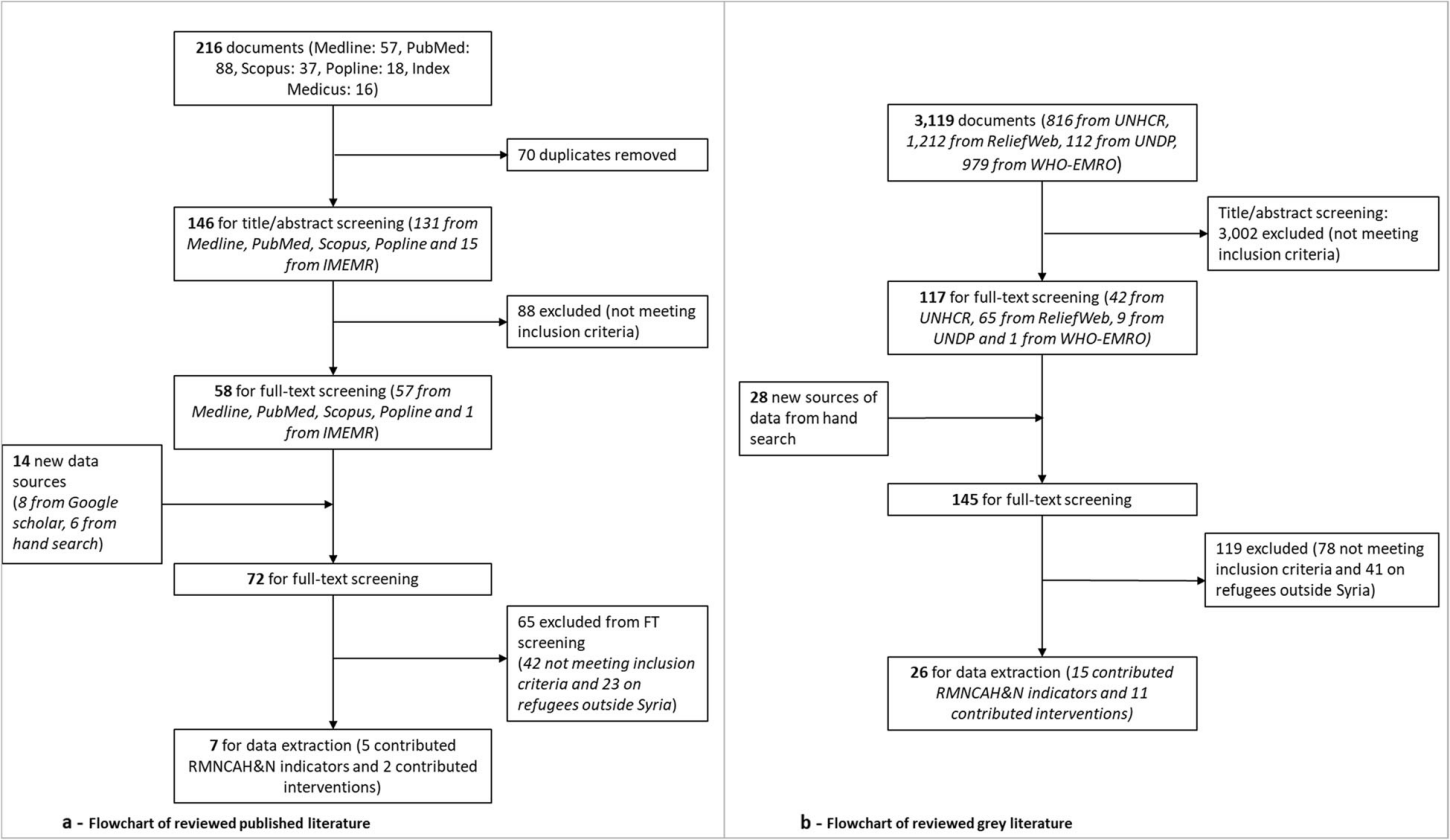
Desk review screening flowcharts

We performed a full-text review of publications that met the eligibility criteria, and extracted relevant data using a standardized KoboToolbox [35] data entry form. Variables that allowed quality assessment (including research design, sampling strategy and sample size, among others; see Additional File 1, Table S1) were abstracted when available in addition to the relevant indicators, interventions implemented, and factors affecting implementation. The screening flowcharts of the published and grey literature reviews are presented in Fig. 2.

The newly extracted indicators from the updated review were merged with the findings of the initial review and summarized in Additional File 1 (Table S2, S3 and S4) by year and geographic region. We mapped the availability/accessibility of RMNCAH&N indicators by geographic region using ArcGIS [36]. Where the data allowed, periodic trends in certain coverage indicators were explored and plotted in the form of line and area charts.

### Key informant interviews

We conducted semi-structured interviews between July and November 2018. We identified the major organisations within the humanitarian emergency response in Syria from the Humanitarian Response in the Whole of Syria. Contact details of cluster and sector leaders were publicly available. A preliminary list of all potential stakeholders (decision makers and health program implementers) was put together and expanded using the snowball technique. We approached 41 potential participants of whom 16 (40%) refused or did not respond, and 25 provided informed consent across the Whole of Syria and the Damascus, Gaziantep and Amman hubs. We sought key informants from UN organisations, international and local NGOs, local health authorities and academics (Table 1).

**Table 1:** Key informants by hub and agency type

	Whole of Syria	Damascus hub	Jordan hub	Turkey hub	Total
**UN agency**	4	3	1	2	**10**
**International NGO**	1	1	5	2	**9**
**Local NGO**			1	3	**4**
**Local health authorities**				1	**1**
**Academia**		1			**1**
**Total**	**5**	**5**	**7**	**8**	**25**

Questions to study participants related to the specific delivery of interventions, the role of the represented organisations with respect to the provision of RMNCAH&N interventions, reasons behind provision/non-provision of certain interventions, rationale informing the decisions, priorities, collaborations and implementation-related questions (Interview guides available in Additional File 3). Only three interviews were held face-to-face while the rest were conducted through telephone/Skype calls, and 16 interviews were conducted in English while the rest were in Arabic. All interviews were transcribed verbatim, Arabic ones were transcribed in Arabic then translated to English and all transcripts were coded by two team members independently using Dedoose 8.1.8 [37].

We analyzed the transcripts of interviews using the latent content analysis approach [38]. This involved an initial reading of interview transcripts from which we developed a preliminary list of key ideas and recurrent themes. Considering the topic guide questions and themes that emerged, we organised the data into categories and identified relationships among and between categories, which allowed us to understand explanatory patterns. Quotes included in the results section have not been attributed to type or location of organisations for reasons of confidentiality.

## Results

### The humanitarian architecture in the Syrian conflict

The nature of the Syrian conflict has shaped the humanitarian response; as the conflict fragmented territories across different governing authorities, it was difficult for the response to have a unified coordination approach. This resulted in a complex coordination and implementation structure under what is termed “The Whole of Syria Approach”. Within this structure, WHO led the health sector/cluster^5^, with reproductive health being co-led by United Nations Population Fund (UNFPA), and with the United Nations Children’s Fund (UNICEF) leading the nutrition sector/cluster. In Damascus, these structures were co-managed with the Ministry of Health (MoH).

*“[In other conflict settings…] there is only one coordinator. In Syria, there are four hubs, and in addition, one of those hubs, the Damascus one, also has three sub-offices where there is also coordination taking place. So in this case, there is quite a multi-layered approach to the coordination.”* (R11).

The UN cluster approach was activated at different times in different geographical regions starting in Damascus as sector coordination in January 2013 [39] and accommodating the new hubs following the UNSC resolution 2165 [20] with Gaziantep in late 2014 and Amman in late 2015. One key informant criticized the delayed establishment of the coordination mechanisms especially in the North.

*“If I were to […] highlight […] how we could have done things differently, the fact that the cluster system was not established until way late into the response, is the failing of the cluster system itself.”* (R14).

The presence of different coordinating hubs was reported to cause difficulties in having a harmonized approach and aligned standards across the four hubs. These were reported to be partly due to the distinct approaches of the NGO/UN-led system versus the existing national system or those established by other governing bodies (temporary government or the Kurdish administration).

“*It’s very hard to align them [the different approaches] because you’re trying to mix a national system with an approach that’s led by NGOs from different sides*.” (R06).

Several representatives, some of whom were of implementing agencies, reported facing restrictions imposed by governing entities as to where they could operate. Organisations operating in government-controlled areas are required to be registered in the country and to work through locally registered organisations such as the Syrian Arab Red Crescent (SARC) and are not allowed to be operational in non-government-controlled areas.

To overcome restricted access to non-government-controlled areas in the North and the South, the humanitarian actors adopted a remote management strategy from the Gaziantep and Amman hubs, which had to be agile by continuously adapting to the prevailing political and security situations affecting the ability to cross borders. Adopted strategies ranged from: i) direct implementation with remote management, whereby organisations were registered and based outside Syria, but sent staff across borders to deliver services; to ii) sub-contracting local NGOs with regular monitoring, and communication through telephone, Skype calls, or visits to Gaziantep or Amman; and iii) supporting local organisations with funding and supplies with no mechanisms to monitor the quality of delivered services.

*“We have five different types of remote programming that we can run. Right now, the remote program you can run inside Syria is the […] one that we don’t want: […] it’s just dumping money with no quality assurance […]*” (R19).

The volatility and dynamism of the political situation in Syria have led to constant shifts in control affecting the sustainability of both operations and coordination.

*“When you look at the maps now, you will see the changes in conflict lines, so when a service was provided by a particular […] service provider, whether private, public, or NGO at the time, and there is a shift in control lines, [service provision] either stops or completely disappears until this is revitalized to a public facility or a private facility that provides services [by organisations on the other side of the conflict]”* (R06).

### Decision making process

Decision making in the Syrian conflict was reported to follow the standard Humanitarian Program Cycle – a process that includes identifying needs, planning activities, implementation, and monitoring and evaluation on a quarterly and annual basis [40]. This process was reported to be centralized at the Whole of Syria coordination level, with the engagement of the various sectors and working groups within each hub. Planning is coordinated with local authorities, and in the Damascus hub, the MoH was reported to be co-leading the health and nutrition responses.

Although no data from the desk review could support this finding, the majority of key informants emphasised that the prioritisation of geographical areas of operations is independent of political governance over the area, and mainly driven by the magnitude of identified needs and the degree of vulnerability of the concerned population [41]^6^.

“*All of our interventions [are] need-based, so we are area blind, we are control blind, we are government blind, and we don’t factor that [in] when we assess the needs*.” (R10).

The massive waves of internal displacement to areas of operations led to constant fluctuations in population size and needs, therefore interfering with the implementation of planned services, and creating a need to monitor the situation on a regular basis (Box 1).

Restricted access was reported by several key informants as one of the challenges to conducting needs assessments and delivering health and nutrition interventions in hard-to-reach areas where the security situation was most severe.

“*You have to coordinate locally and seek approvals of the security apparatus and the system that works. It’s a bit challenging, you have to work with [these]authorities, in identifying the needs and seeking approval [to access] those areas.”* (R12).

Humanitarian organisations engaged in successful negotiation and coordination efforts with local authorities allowing them to gain access to hard-to-reach locations.

*“There was a small Polio outbreak in Deir-ez-Zor, which is a marginal area in the North East, [so] there had to be negotiation with [opposition parties] to actually run a Polio [vaccination] campaign in that area. And it was the Ministry [of health] who accepted as well. […] It was a very strong negotiation to make sure that the response was implemented, which I think was an amazing success story”* (R06).

In addition to population needs, and according to some key informants, intervention delivery was reported to depend upon partners’ technical and financial capacities to implement, whereby a decision maker stated to have formally evaluated these capacities through the “Organisation Capacity Assessment”.

“*[Sector leads] highlight all priorities, and then we see who’s willing to take what, depending on their capacity […], sometimes we just go to a certain partner because we know that this partner has the capacity to do this. And sometimes they would take the offer […] and sometimes they just decline.*” (R01).

It was reported that the entire civil society system in Syria faces operational challenges. Pre-existing NGOs were not accustomed to functioning in emergency circumstances, as opposed to other contexts at the international level that had suffered from conflict, and where the humanitarian response has existed for decades.

*“I believe, [in other settings] we’ve been having emergencies since existence began, so the NGO capacity has been on-going for the last 25-30 or 40 years, but Syria was pretty much a stable country until this particular [conflict]. So, the issue of having NGOs and how NGOs are run, the whole operations, is also [challenging], so there is a limit to what you can expect.”* (R02).

Nevertheless, a number of operating partners in Syria reported that they compensated for the shortage in capacities by complementing each other’s activities on certain occasions, thus ensuring the implementation of essential programs.

“*For example, [if] we have nutrition surveillance and IYCF [program capacity], and there is [an]other partner [that] can provide surveillance in this [geographic] area, […] then we will only provide IYCF. The full package is to provide both, but we can depend on other partners.”* (R09).

The Humanitarian Response Plan also depended on the availability of funds and their allocation. In some instances, donors’ political agendas were reported to influence access and intervention delivery to certain geographic locations.

“*[Donor X] will not agree to carry out an intervention in specific areas where some specific groups are in control. By these groups, I mean the radical ones. However, the civilians there are in need […] but no intervention can be carried out.*” (R21).

Similarly, and as the crisis progressed, there was a reported shift from “*un-earmarked*” funding to donors enforcing restrictions on the types of funded activities, which influenced the spectrum of available services. For example, one donor had a constraint on procuring contraceptives, which hampered the provision of family planning services.

*“[Donors] influence the agenda because they have the money. If they don’t want to support or fund something, this is not going to happen even if you advocate for that thing for a zillion year.”* (R01).

Moreover, donor fatigue and the multiple competing crises in the world over a single pool of financial resources have contributed to a reported shortage in allocated funds. The health and nutrition responses had to adapt by compromising certain planned interventions or prioritising certain ones over others. Resource limitations were cited to influence, for example, the ability to provide neonatal intensive care.

“*We also focused on [newborn intensive care], but, [due to] the lack of resources and funding, we could not treat it as a top priority*” (R15).

### Factors that influenced intervention coverage and adaptations

Three key factors were identified as influencing RMNCAH&N intervention delivery, coverage and adaptations in interventions: (1) prioritisation (or the lack of thereof) of RMNCAH&N interventions, (2) the security situation, which influenced health seeking behaviors of populations in this context, and (3) a mismatch between interventions provided by the humanitarian response and national in-country practices existing pre-conflict.

#### Prioritisation (or the lack of prioritisation) of RMNCAH&N interventions

As the crisis progressed and needs intensified, service prioritisation by humanitarian actors was reported to influence the types of interventions delivered and their coverage. Child vaccination was delivered as one of the first-line interventions with the MoH leading the response from Damascus, but differences in geographical coverage remained (see Box 2). The early response also focused on injury care and providing food and shelter. However, humanitarian agencies were able to successfully advocate for prioritisation of reproductive and maternal healthcare, such as antenatal, delivery and postnatal care based on evident needs. Family planning was reported to have been overlooked in the early phases of the response, and took time to be re-established, with certain reported geographic discrepancies.

*“Family planning services were not very strong in the public facilities in the first place. […] Before the crisis [family planning] was one of the […] mandates of an NGO that had very strong reach […], I don’t think there was a focus on family planning in the emergency mode […]. [The response] took some time to re-establish […] When you have a facility in a location, it might have stopped for a while, it might have changed locations, your staff might have completely disappeared; […] [family planning services] slowly over time sort of revitalized”* (R06).

Delays in establishing family planning services were also due to certain donors refusing to fund the service, and governing authorities restricting service delivery in certain geographic areas. Whereas in other areas, donors pushed for family planning programs.

It appears that, as was the case for family planning, services that were reportedly not strongly institutionalized in the public sector pre-conflict were also those that were not prioritised in the emergency humanitarian response. Another example is that of adolescent-specific services, which remained lacking despite the presence of early marriage in Syria and the recognized need to therefore target this age group.

*“Based on what we’ve seen, we tried to initiate some kind of messaging around the dangers of early pregnancy, and delaying the age of marriage, but we’re not spanning the full spectrum of adolescent SRH at the moment”* (R14)*.*

A few key informants also reported that the low prevalence of malnutrition (Additional File 1, Table S4), in addition to the lack of data and evidence on infant and young child feeding (IYCF) indicators contributed to delaying the nutrition response.

#### Security influenced health seeking behaviors

As coverage rates of certain interventions decreased during the conflict (e.g. contraceptive prevalence rate), and data on others were completely lacking (e.g. ANC 4+ (at least four antenatal care visits) and PNC) (Additional File 1, Table S2), humanitarian agencies implemented outreach and awareness raising activities to promote and extend the availability of services to beneficiaries. Despite these efforts, insecurity limited women’s movement and populations feared violent attacks, including attacks on healthcare facilities.

*“Although we are offering all these services [safe delivery] at the hospitals and comprehensive primary health centers, the security issue is limiting pregnant women’s access to the centers in fear of being attacked. Because […] there is a systematic targeting of health centers and hospitals including obstetric and women hospitals. […] This means that there’s still a high proportion of home births that we cannot determine. Thus, we are implementing something called a community midwives [program].”* (R18).

The community midwives program, which was reported to be implemented in the Northwest, given Syrian women’s reported preference for home births, ensured skilled birth attendance whereby birth registration was reported to be conditional upon a trained midwife’s signature of required paperwork. Midwives were also trained to refer complicated deliveries to hospitals; this was one of the factors contributing to the increasing facility-level C-section rate in Syria during the conflict, while the population-level indicator remained more-or-less constant based on the findings of the desk review (see Box 3 for additional details).

#### A mismatch between humanitarian interventions and previously existing practices

Several respondents referred to a mismatch between interventions proposed by humanitarian agencies and previously existing national programs and practices. In addition to the newly-introduced community outreach activities mentioned above, other examples include procurement of certain vaccines such as the pneumococcal conjugate vaccine antigen, which proved to be difficult as it is not part of the national Expanded Program on Immunization (EPI) schedule in Syria [42]. Several key informants also stated that the health and nutrition responses focused on the delivery of the essential, life-saving, cost-effective primary healthcare packages, such as the Minimum Initial Service Package (MISP), Integrated Management of Childhood Illness (IMCI) which existed previously in Syria since 2000 [43], and nutrition surveillance. However, this modality of delivering healthcare was resisted by Syrian beneficiaries at first due to the reported pre-conflict pattern of by-passing primary services and specialist visits being the first point of contact.

*“When we started off, […] it was a bit difficult to convince the general populations to seek services at primary healthcare facilities. This is because their mindset was that ‘when I get sick, I go to a hospital and I see a specialist.’ […] Over the last five years or so, I guess that mindset has changed a little, and the fact that primary healthcare is still quality healthcare, that concept has slowly sunk in.”* (R14).

The humanitarian response did, however, evolve over time, and as priorities were being met there was more opportunity to deliver second- and third-line interventions. For example, within the nutrition sector in Damascus, it was described how once programs for the treatment of acute malnutrition were well-established, organisations were then able to move to supporting the baby-friendly hospital initiative. Furthermore, few key informants explained that actors started to address issues related to quality of care later on during the crisis.

*“At the beginning, […] you would go and provide life-saving activities depending on how many beneficiaries you served. Now, it’s different. You have to identify exactly what the activities are that you want to provide based on a needs assessment, […] what protocol you want to apply, and what’s the indicator that will reflect the impact of your intervention. So this way we add more quality to the intervention.”* (R09).

### Factors that influenced the modality of service delivery and adaptations

#### Pre-conflict Syria: a middle-income country with existing health capacities

Pre-conflict Syria was a middle-income country with adequate healthcare delivery capacities, and few key informants highlighted the importance and need to adapt humanitarian intervention to make use of pre-conflict skilled capacities.

*“I think that [the] Syria crisis has challenged the humanitarian community, […] Syria was a middle-income country, [while] the humanitarian community is mostly used to low-income countries. So, we cannot just say OK this is what we’ve done in other emergencies and this is what we do in Syria. […] We need to think more creatively and adapt [better] to the local context because we have capacities in Syria.”* (R16).

*“Paediatricians are competent, and they have degrees. You cannot come and tell them to go back to the level of IMCI. […] The situation here prior to 2011 was advanced compared to other countries that have emergency zones…”* (R18).

The health infrastructure did, however, partially collapse and the humanitarian response then built on the previously existing infrastructure by rehabilitating primary and secondary healthcare facilities, supporting them financially or through in-kind assistance, and/or recruiting staff in order to ensure free access to services by populations in catchment areas.

#### Shortage in specialized healthcare providers

The conflict also led to a large exodus of skilled healthcare providers, which necessitated a task-shifting strategy. This was achieved by training general practitioners, surgeons, nurses and midwives to fill the gap in specialized healthcare workers (mainly obstetricians/gynaecologists), instead of recruiting non-local healthcare professionals (Box 2). However, the quality and efficacy of the capacity building was reported to be questionable by their initiators themselves in the context of remote management, as training was conducted through phone or Skype calls with limited possibility for hands-on applications.

*“[Health workers] just received a bunch of scattered, random trainings from different organisations, and they became psychosocial workers. It happened also in nursing, midwifery and other specialties, and this was another challenge we faced in terms of confirming credentials of staff”* (R01).

Teams of healthcare staff were also required to rotate amongst different facilities to fill the human resource gap.

Implementing organisations faced difficulties in recruiting skilled female practitioners, such as gynaecologists, pediatricians (particularly among rural women) and nutrition specialists, who were reported to be preferred by female service users. This was especially true in the nutrition sector/cluster where the modality of service delivery entailed household visits; this could have been due to security concerns or cultural norms.

“*The majority of the interventions they were planning to conduct [in the nutrition sector] consisted of household visits and day visits. […] People would not let [young men] into their houses since, during the day, there are only women in the house*.” (R08).

The challenge of recruiting skilled female practitioners was further exacerbated by the preference of trained health workers to practice in large cities.

“*It’s very difficult to find a pediatrician. They usually want to stay within the larger cities, and to have their own private practices instead of work with an NGO in a camp setting.*” (R14).

#### Displacement of populations

The massive waves of internal displacement of populations also led to interruptions in the continuity of care and follow-up visits for displaced beneficiaries.

“[*Displacement] is interrupting the [continuity] of services. It is actually fragments of activities happening in different places, in different times, by different people*” (R10).

IDPs might not always be aware of the facilities where they can seek health services. Thus, mobile units were activated to respond to this need. Mobile health teams provide basic RMNCH&N services and consultations to IDP sites and hard-to-reach areas and are considered entry points for any needed referral to other primary or secondary care levels. These were complemented by “convoys”, which are mainly used to provide one-off services such as the distribution of reproductive health kits or nutrition supplements.

To access hard-to-reach areas, community outreach and awareness raising activities were conducted.

*“The gap of that system was in the hard-to-reach areas because we couldn’t apply the same method, so it was more of outreach, you had to try to make contact with people inside those blocked areas.”* (R06).

“*I will tell you about one specific […] hard-to-reach area that we supported. We send them the trucks of the drugs, at night with the car’s and the truck’s lights off. The truck moves slowly in order not to produce any sound*.” (R21).

#### Procurement restrictions

While key informants did not report concerns over stock availability of reproductive health related items, there were reported restrictions on procuring certain supplies. Thus, implementing agencies had to use commodities available in the local market despite their uncertainty regarding the quality of local supplies.

*“[There were] several restricted items, […] mainly the ketamine, which had direct influence on reproductive health. This is an anesthetic, […] and it’s preferred in surgeries and maybe caesarean sections, so with high rates of C-sections that we had in the South, […] mostly if [ketamine is] available, it’s from the local black market with very questionable quality*.” (R01).

Few key informants also reported that the purchasing process was too lengthy and interfered with the ability to provide urgently needed services. To overcome the bureaucratic procurement process, some UN agencies were asking implementing partners to purchase supplies since *“[they] have a little bit of flexibility; [their process] is faster than ours*” (R02).

#### Attacks on healthcare facilities

Targeting of healthcare facilities and ambulances and kidnapping of healthcare workers was reported across all hubs as a challenge. Many implementing partners developed patient evacuation plans and contingency plans in case of attacks on healthcare (e.g. operating in secondary locations, service decentralization, and fortification of health facilities). On the other hand, some facilities completely refused the support of NGOs as receiving NGO support was perceived to increase the facilities’ risk of being attacked.

The choice of health facilities by implementers was also reported to depend on their locations to minimize the security risks on populations attending the facilities. Humanitarian actors mostly chose small size health facilities, or “*underground, or secured, fortified facilities*” to mitigate the risk of targeting, and to prioritise the safety of patients and health staff.

*“One of the considerations for where [to open a facility] has always been that we have specifically looked at facilities that were not at direct risk of being close to […] a military output or something like that where the population coming to the facility would be at risk.”* (R14).

Box 1 – RMNCAH&N data availability In the setting of Syria in conflict, data collection, reporting and data availability are complicated processes. Political constraints limit the scope and detail of information that can be reported and shared by the health system and humanitarian actors. Our desk review and interviews reveal glaring gaps in data on certain RMNCAH&N indicators that could be the result of either lack of data or issues with data sharing. Examples include data on maternal mortality ratio, proportion of women attending 4 or more ANC visits, proportion of women accessing PNC, prevalence of low birthweight among newborns and indicators related to adolescent health.

Certain key informants perceived these gaps to be the result of the absence of a “*unified health information system”* in the country; non-compliance in reporting from implementing partners; and difficulties in conducting population-level assessments and surveys given the security situation and remote management.

“*Here, we have not put [the surveillance of maternal deaths] in place, […] and the reason [for that] is because of the remote [management], [I] could not go on the ground, so I cannot work with audit teams so that they work in a proper manner, and I felt that there was too high a risk of damage, of harm, in setting such a team without training them before*.” (R07).

In addition, similarly to the findings of DeJong et al.’s review, data on certain indicators were not accessible to the research team although they were known to be available [24]. According to several key informants, this is the result of organisational policies that restrict data-sharing, and the nature of the assessments, which are usually internal and performed to inform planning and therefore there is a reluctance to share them. Similarly, while a strong health monitoring system is in place in public Syrian health facilities (HeRAMS), access to the raw data is limited, and only reported aggregate measures could be used. Moreover, the value of such a system is constrained, as it cannot be used to generate population level indicators, since the healthcare facilities’ catchment population cannot be reliably estimated nor disaggregated by age and gender, especially considering the extremely dynamic context [6, 44].

Even when data are available, their validity, quality, and representativeness cannot be ascertained. One key informant highlighted how the dynamic nature of the context – both in terms of population displacement and frequency of armed conflict events – reduces the validity of the data over a long period, particularly considering the ever-changing needs. Furthermore, data quality was reported to be threatened by the remote modality of operations.

*“At the end of the day, whatever data we receive, […] we don’t have any method to validate this data, […] we always have this kind of leap of faith, so no matter what kinds of measures we take in place, at the end there is a blind space […]*” (R01).

In an attempt to adapt to the security constraints, agencies were reported to be using unconventional methods to collect quantitative data, such as conducting rapid appraisals with local community members. Although the reliability of these assessments and the resulting data quality are uncertain, they remain a feasible option for obtaining information in such a current volatile context [6]. Yet, this led to reported indicators not being consistently defined in published reports/papers, and not being consistent with internationally agreed upon definitions (e.g. immunization coverage measured in different age groups, or different dosages; reporting crude numbers of those who received the service instead of proportions; Additional File 1).

**Fig. 3:**
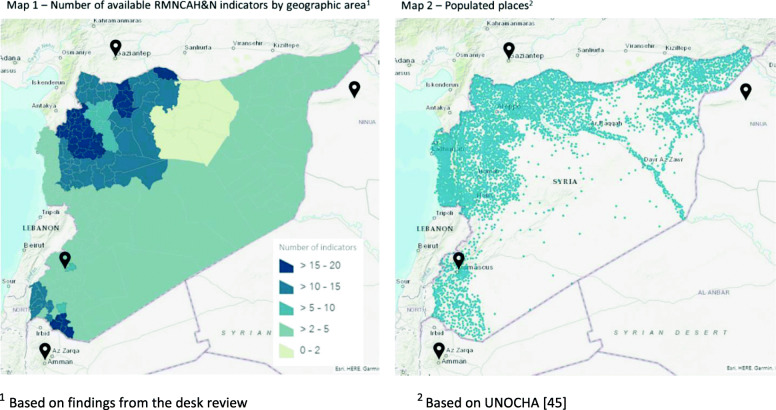
Maps of RMNCAH&N indicators by geographic area and populated places

Map 1 of Fig. 3 displays the number of RMNCAH&N indicators (of those listed in Additional File 4) collected from the desk review by geographic area, whereas Map 2 displays populated areas in Syria as per the United Nations Office for the Coordination of Humanitarian Affairs (UNOCHA) [45]. These highlight the fact that there are several densely populated Syrian territories on which RMNCAH&N data were not accessible/available in the published and grey literature. Key informants additionally reported that published indicators are not nationally representative as the humanitarian response does not report on government-controlled areas.

“*Let’s not forget that in vast areas, the health system is still intact, Ministry of Health, Ministry of Higher Education… so we’re not reporting what’s happening all over health, all over Syria, by everyone*” (R10).

**Fig. 4:**
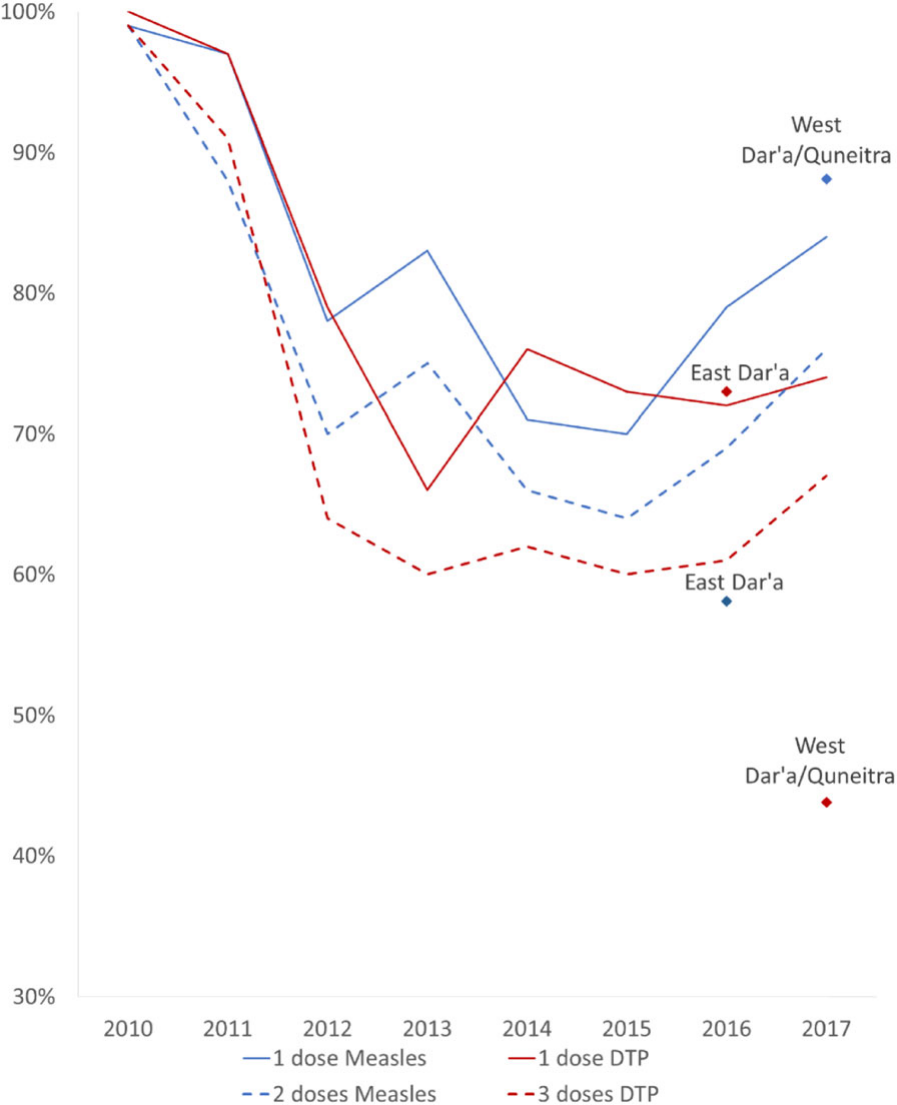
Measles and DTP vaccination coverage rates across Syria in West Dar’a/Quneitra [46], East Dar’a [47], as compared to national WHO-UNICEF data [48], from 2010 to 2017

Box 2 – Immunization The partial collapse of the healthcare system during the conflict led to disruptions in the vaccination schedule, and coverage rates decreased with time (Fig. 4), leading to the re-emergence of Polio in 2013 [49]. However, the national communicable disease surveillance system was strengthened during the war, as the MoH, supported by WHO, established an Early Warning Alert and Response System (EWARS) [50]. Together with Early Warning Alert and Response Network (EWARN), which was functional in opposition-controlled areas, surveillance was successfully maintained and able to detect and control multiple outbreaks, including Polio [51]. Throughout the conflict, the Syrian MoH was leading the immunization response in Damascus and the surrounding government-controlled areas, as well as in the South where humanitarian agencies would report gaps in coverage to MoH teams. On the other hand, in Northern non-government-controlled areas the Syria Immunization Group has coordinated the immunization response since 2015.

Figure 4 displays trends in official immunization coverage rates across Syria from 2010 to 2017 for measles and DTP [48]. As the response evolved from supporting immunization campaigns to strengthening the routine immunization program, rates increased to over 70% coverage with time; yet they remain suboptimal due in part to gaps in resources, and inconsistencies in providing services. The result is that in certain areas coverage of the first dose remains higher than coverage of subsequent doses (Fig. 4).

*“The message is not that no vaccinations happened or that there is a huge pool of unimmunized children, but that there are under-immunized kids, who are just off schedule because of war disruption*” *(*R01).

In South Syria, there were evident geographical discrepancies regarding antigen-specific coverage rates. Data were available for Dar’a and Quneitra at two time points [46, 47]. While East Dar’a had higher coverage of the DTP vaccine, which is administered through routine immunization, it had lower rates of the measles antigen, compared to West Dara’a and Quneitra. This was attributed to the different access/security situations in these two regions, which led to adopting different modalities of vaccination provision. In East Dar’a, there was ongoing communication with the MoH, and the EPI was provided in public vaccination centers. However, this was not the case in West Dar’a/Quneitra, where humanitarian agencies conducted two cross-border measles and polio immunization campaigns to this region in 2015 and 2016. It was also mentioned that the small population size in Quneitra kept it epidemic-free.

“*It had so many fewer people that, your pockets of under-immunized kids are probably smaller than even one large town in the whole governorate*” (R01).

Box 3 – Delivery care and caesarean-sections The availability and modality of emergency obstetric care varies across Syrian territories. In regions where Comprehensive Emergency Obstetric Care (CEmOC) is available and where the security situation is relatively stable, caesarean sections (C-sections) are commonly performed, to the extent that the operation was perceived as a “*habit, a trend, more than really it should have been, more than the necessary*” in certain regions (R06).

**Fig. 5:**
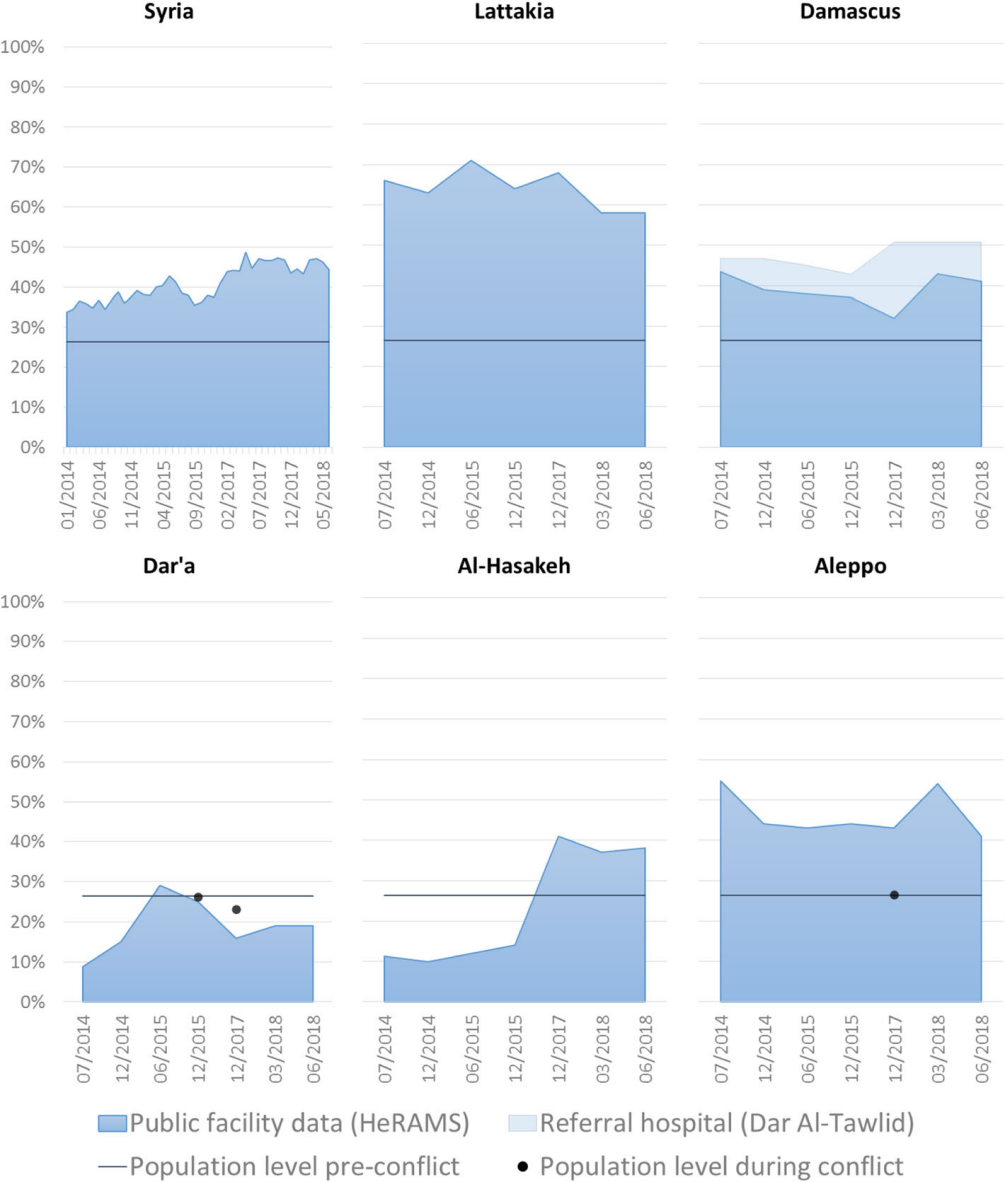
C-section rate during the Syrian conflict in healthcare facilities [33], Dar Al-Tawlid Hospital [52], and at the population level [47, 53, 54] at the population level pre-conflict [55]

In fact, there was consensus among key informants from all hubs that the proportion of deliveries by C-section increased during the conflict. Key informants from the South reported rates reaching an average of 40% at facility level, while those based in Damascus reported a rate of 80% in the private sector. These rates were also reported to fluctuate across areas ranging from 10% in North-East Syria to around 48% in Hama. Figure 5 shows the evolution of C-section rates at public health facilities reporting to HeRAMS over time, both on the national level and in five selected Syrian governorates (Lattakia, Damascus, Dar’a, Al Hassakeh and Aleppo), in addition to population-level estimates retrieved through the desk review (Dar’a and Aleppo), and national pre-conflict population-level C-section rate [55]. The graph on Damascus also includes data from Dar-Al-Tawlid, a public maternity hospital [52].

Several key informants reported numbers based on situation assessments they contributed to or on anecdotal knowledge, and these reported C-section rates were aligned with numbers in Fig. 5. On the other hand, population level estimates of C-section rates are lower given the high proportion of home deliveries, which range from 13% in Aleppo and Idleb [53] to 29% in Eastern Dar’a in 2017 [54].

“*Cases that do come to hospitals are those that require a C-section because this can only be performed at the hospital”* (R18).

Additionally, the security situation and the targeted attacks on healthcare facilities have pushed women to opt for a scheduled C-section. One study in 2017 has shown a significant positive association between war events (explosions and clashes) and the number of C-sections [56].

“*Women would show up and say: ‘I want a C-section now because I don’t want to stay in hospital […] I am afraid it’s going to get attacked’, and so why would I [the woman] sit here in labor for 20 hours and give 20 hours of vulnerability to an attack?’*” (R01).

Moreover, four key informants noted that high C-section rates can be attributed to healthcare providers’ interests in time management and financial gain, particularly among those who would refer patients to their private clinics.

“*Physicians and obstetrics/gynecology specialists become more, ‘ok let me try to make sure that I have my time organised’ […] doctors are no longer patient. For some of them, not for everyone, this has unfortunately become a commercial process*.” (R13).

Following the exodus of skilled obstetricians/gynecologists, task shifting of delivery care to surgeons has also contributed to the increase in C-sections, since “*a surgeon is not an obstetrician, so for them the standard of care is ‘the best, the safest, […] thing for me to possibly do is to give a woman a C-section, cut and go’*” (R01).

Few key informants also reported that since before the conflict, Syrian women have preferred seeking care at hospitals instead of delivering at primary healthcare centers. This is in line with the findings of Bashour et al. which show that most Syrian women preferred to give birth at hospitals because they perceived it to be safer than home births [57]. Some key informants also pointed out that women may choose to undergo a C-section, a procedure that could lead to increase the rates of potentially dangerous repeat C-sections in the future, instead of a normal birth. In Lebanon for example, 57% of C-sections among Syrian refugees were attributed to repeat C-sections during the first half of 2013 [58].

Nonetheless, in 2018, efforts started to be put in place to reduce C-section rates within Syria, including by the MoH and professional bodies who are leading discussions on this practice and its implications. Other activities involve advocating for safe and rational delivery care among implementing partners and raising awareness among beneficiaries to encourage normal delivery. However, achieving this target was perceived to be complicated and challenged by the lack of time and remote management.

## Discussion

Before the conflict, Syria was covered by an accessible and extensive public healthcare system, in addition to a medicalized pattern of care with frequent recourse to specialist care. As the war occurred with its shifting control lines, high-level decision making, the healthcare system, health workforce and the modality of service delivery rapidly changed. It is noteworthy that the humanitarian response was not specific to RMNCAH&N but rather encompassed all health services. Those services that rely on a stable health system, such as immunization, suffered, and it took some time for the humanitarian system to establish itself and achieve some progress.

Our findings suggest that the partial breakdown of the healthcare system and the delay in humanitarian coordination led – at certain times, and in certain geographic locations – to gaps in intervention delivery and coverage along the continuum of care for women, children and adolescents. This lag raises concerns regarding the lack of emergency preparedness in a wider region that has been particularly prone to conflict. Resulting gaps within Syria varied by region and time in this particularly heterogeneous setting, in terms of political governance, functionality of healthcare systems, security, and accessibility. This led to fluctuations in family planning services, newborn care, IYCF programs and immunization coverage. Additionally, a lack of focus on adolescent health was identified throughout the conflict.

The volatile security context led to i) population displacement, thus interrupting the continuity of care for concerned populations; ii) exodus of skilled health workforce leading to a shortage of healthcare providers following recurrent attacks on healthcare facilities; iii) enforcing sanctions on procurement of supplies [59] and iv) restrictions on population movement, and thus access to healthcare. These factors combined have affected healthcare seeking behaviors, which is exemplified in the case of delivery care, whereby Syrian women were reported to prefer home births, or request C-sections to mitigate the risks associated with the insecure environment, thus – combined with provider incentives – leading to increasing rates of facility-level C-sections.

This study also highlights that whereas maternal and child health interventions were prioritised by humanitarian actors from the very start of the crisis, the delayed prioritisation of certain RMNCAH&N interventions (such as those related to family planning and adolescent health) has affected intervention coverage. This is in line with findings of another study from inside Syria, which found that the initial response prioritised trauma care and infectious disease control [60]. Garry et al. focused on non-communicable disease care provision and found that priorities were donor-driven as opposed to population needs-driven [60]. Our findings also illustrate that services not prioritised in Syria’s public health system pre-conflict (e.g. family planning services) were also not initially prioritised by humanitarian actors in their RMNCAH&N programming. It is however interesting to note that certain components of the health system, such as communicable disease surveillance, retained some level of functionality and enabled an informed response to the detected outbreaks.

The humanitarian response mainly focused on investing in delivering care at primary healthcare facilities, which is contrasted with how the population sought care pre-conflict (e.g. with specialists at hospitals). Key informants reported that over time, the Syrian population started to value the importance of health care services at the primary healthcare level. These findings are in line with those from the study focusing on the delivery of non-communicable diseases care inside Syria [60]. The Syrian population’s early resistance towards the less medicalised care model offered by the humanitarian system suggests that the latter should better align with the pre-existing health system in a conflict-affected setting. Doing so necessitates a rapid health system assessment of the context before starting service delivery, and ongoing communication and coordination with local stakeholders, which is a challenge in the Syrian context where political instability restricts humanitarian actors’ operations and movements [61].

The fragmentation of governance on the Syrian territory among multiple authorities led to a multi-hub response [6] that is unique to Syria. While this complex humanitarian architecture has implications on the modality of service delivery, and therefore coverage, it allows the successful provision of RMNCAH&N interventions in a context with fragmented governance. Despite the reported challenge regarding fluctuations in border crossings, the adopted remote management strategy has permitted humanitarian agencies to expand their reach, and serve populations in need of healthcare in often inaccessible areas [61]. Similarly, the newly introduced modality of service delivery entails an increased emphasis on outreach activities through community health workers providing health education, home visits and health screening. This approach was not part of Syria’s pre-conflict health system, and has positively influenced RMNCAH&N service delivery, and potentially the coverage of such interventions.

Acknowledging that remote management was considered to be a key operational solution, we identified potential limitations of this modality in ensuring coverage and quality, which should be taken into consideration in the adoption of a similar modality. These were largely related to the lack of robust accountability and monitoring mechanisms, which hinders the assessment of the quality of delivered interventions and the validity of collected data [62]. This deficiency in accountability was reported to be one among several constraining factors (e.g. measurement difficulties) that have prevented the establishment of a maternal mortality surveillance, leading to a knowledge gap in the Maternal Mortality Ratio (MMR) indicator in Syria during the conflict. Other gaps in RMNCAH&N indicators were also documented through our current desk review, which are consistent with the findings of our earlier study [24]. Although we could retrieve data on the regional variation in immunization coverage and C-section rates, we are not able to present geographical disparities on other RMNCAH&N indicators due to the above-documented data gaps and their underlying causes.

Our analyses also highlight the lack of representative data at all levels (i.e. national, governorate and sub-governorates levels) as well as reliable population-level data, and pre-conflict benchmarks for certain indicators (e.g. facility-level C-section rate), in line with other studies [6, 24]. We relied on published data sources due to our inability to obtain raw data from various sources (including, for example, HeRAMS). This limits the representativeness of the analyses, particularly as data sources such as HeRAMS include data only from public healthcare facilities and may also suffer from incomplete reporting [33]. Vaccination data that was used also relies on published sources that have evaluated the quality of data as low [48].

### Strengths and limitations

The strengths of this study lie in its methodological approach, which used triangulation and validation across qualitative and quantitative data to better describe the factors that influenced RMNCAH&N intervention coverage in the Syrian context where data quality and quantity are limited. Furthermore, this research was conducted by a team the majority of whom are based in a neighboring country and thus are familiar with the context. However, our study was limited by the difficulties of the Syrian context. We had restricted access to Syrian governmental authorities or representatives from local NGOs in Damascus. Thus, although UN informants reported on the MoH collaboration and efforts, we may not have provided a comprehensive picture of services provided by this entity. We were also unable to interview first-line healthcare providers due to the interviews being conducted remotely. In addition, we faced reluctance from some organisations/individuals to communicate with the research team, possibly due to changes in political control of different territories occurring during the study period (which continue to occur). Finally, we were not able to compare the response between the geographic areas controlled by different entities due to the paucity of RMNCAH&N indicators and interventions data at the governorate and/or sub-governorate levels.

## Conclusions

The case of Syria provides a unique perspective on creative ways of managing humanitarian interventions in order to serve populations in need, in a dynamic and often volatile political and security environment. Adaptations made to the humanitarian architecture in the management of the response to the Syrian crisis, specifically the adoption of remote management, can offer potential solutions to manage and deliver RMNCAH&N services in similar conflict-affected settings. Despite operational, human resource, and funding challenges, humanitarian actors were able to prioritise services for maternal and child health (albeit a lagged response); however, a glaring gap remains in the delivery of adolescent health-related services. Furthermore, the scarcity and sensitivity of RMNCAH&N data in the Syrian context has been a tremendous challenge for both researchers and implementing agencies, and these data are needed to hold humanitarian actors to account, with an ultimate aim of improving the health of women, children and adolescents in Syria.

## Supplementary information

**Additional file 1.** Desk review findings.

**Additional file 2.** Desk review search strategy and inclusion criteria.

**Additional file 3.** Key informant interview guide.

**Additional file 4.** List of RMNCAH&N indicators used to build Map 1.

## Data Availability

The datasets used and/or analyzed during the current study are available from the corresponding author on reasonable request.

## Abbreviations

ANC: Antenatal Care
BRANCH: Bridging Research & Action in Conflict Settings for the Health of Women & Children
C-section: Caesarean-section
CEmOC: Comprehensive Emergency Obstetric Care
EPI: Expanded Program on Immunization
EWARN: Early Warning Alert and Response Network
EWARS: Early Warning Alert and Response System
HeRAMS: Health Resources and Services Availability Mapping System
IDP: Internally Displaced People
IMCI: Integrated Management of Childhood Illness
IYCF: Infant and Young Child Feeding
MDGs: Millennium Development Goals
MISP: Minimum Initial Service Package
MMR: Maternal Mortality Ratio
MoH: Ministry of Health
NGO: Non-Governmental Organisation
PNC: Postnatal Care
RMNCAH&N: Reproductive, Maternal, Newborn, Child, Adolescent Health and Nutrition
SARC: Syrian Arab Red Crescent
UN: United Nations
UNICEF: United Nations Children’s Fund
UNFPA: United Nations Population Fund
UNOCHA: United Nations Office for the Coordination of Humanitarian Affairs
WHO: World Health Organisation

## Footnotes

1 MDG4: Reduce child mortality; MDG5: Improve maternal health

2 Informal settlements consist of groups of tents/shelters established by IDPs themselves

3 This resolution allows UN agencies and their implementing partners to deliver humanitarian assistance from neighboring countries using approved border-crossings

4 Bridging Research & Action in Conflict Settings for the Health of Women & Children

5 The term “sector” refers to discrete technical areas of humanitarian action in Syria following the Cluster approach but where the Government is co-managing the coordination. In non-government-controlled areas, Clusters report to the Humanitarian Coordinator without governmental involvement.

6 The magnitude of needs and vulnerability level in a given area is measured using a severity score, which is a composite needs index, with scores ranging from 0 to 6, used for priority setting and planning

## References

[1] Devakumar D, Birch M, Rubenstein LS, Osrin D, Sondorp E, Wells JCK (2015). Child health in Syria: recognising the lasting effects of warfare on health. Confl Heal.

[2] Sami S, Williams HA, Krause S, Onyango MA, Burton A, Tomczyk B (2014). Responding to the Syrian crisis: the needs of women and girls. Lancet.

[3] United Nations Office for the Coordination of Humanitarian Affairs (UNOCHA). Humanitarian bulletin - Syria [11–24 December 2012]. 2012.

[4] Akbarzada S, Mackey TK (2018). The Syrian public health and humanitarian crisis: a ‘displacement’ in global governance?. Global Public Health.

[5] Aburas R, Najeeb A, Baageel L, Mackey TK (2018). The Syrian conflict: a case study of the challenges and acute need for medical humanitarian operations for women and children internally displaced persons. BMC Med.

[6] Diggle E, Welsch W, Sullivan R, Alkema G, Warsame A, Wafai M (2017). The role of public health information in assistance to populations living in opposition and contested areas of Syria, 2012–2014. Confl Heal.

[7] Ben Taleb Z, Bahelah R, Fouad FM, Coutts A, Wilcox M, Maziak W (2015). Syria: health in a country undergoing tragic transition. International Journal of Public Health.

[8] Alzoubi Z (2017). Syrian civil society during the peace talks in Geneva: role and challenges. New England Journal of Public Policy.

[9] United Nations Development Programme (UNDP). Syrian Arab Republic - Third National MDGs Progress Report. 2010.

[10] Kherallah M, Alahfez T, Sahloul Z, Eddin K, Jamil G (2012). Health care in Syria before and during the crisis. Avicenna Journal of Medicine.

[11] Fouad FM, Sparrow A, Tarakji A, Alameddine M, El-Jardali F, Coutts AP (2017). Health workers and the weaponisation of health care in Syria: a preliminary inquiry for the lancet–American University of Beirut Commission on Syria. Lancet.

[12] Safeguarding Health in Conflict Coalition. Impunity must end: Attacks on health in 23 countries in conflict in 2016. 2017.

[13] Haar RJ, Risko CB, Singh S, Rayes D, Albaik A, Alnajar M (2018). Determining the scope of attacks on health in four governorates of Syria in 2016: results of a field surveillance program. PLoS Med.

[14] World Health Organization Regional Office for the Eastern Mediterranean (WHO-EMRO). WHO Response to the Conflict in Syria - Situation Report #4. 2014.

[15] United Nations Department of Economic and Social Affairs - Population Divison. World Population Prospects: The 2017 Revision. 2017. Available from: https://population.un.org/wpp/DataQuery/.

[16] United Nations Office for the Coordination of Humanitarian Affairs (UNOCHA). Syrian Arab Republic Humanitarian Needs Overview 2018. 2017.

[17] United Nations High Commissioner for Refugees (UNHCR). 2017 Annual Report - Regional Refugee & Resilience plan 2017–2018 in response to the Syria crisis. 2017.

[18] Doocy S, Lyles E, Delbiso TD, Robinson CW (2015). The IOCC/GOPA study team. Internal displacement and the Syrian crisis: an analysis of trends from 2011–2014. Confl Heal.

[19] United Nations Population Fund (UNFPA). Syria Humanitarian Emergency - Humanitarian Needs. 2017.

[20] United Nations Security Council Resolutions. Resolution 2165. 2014. Available from: http://unscr.com/en/resolutions/2165.

[21] United Nations Office for the Coordination of Humanitarian Affairs (UNOCHA). Humanitarian Response - Whole of Syria. Available from: https://www.humanitarianresponse.info/operations/whole-of-syria.

[22] Douedari Y, Howard N. Perspectives on rebuilding health system governance in opposition-controlled Syria: a qualitative study. Int J Health Policy Manag. 2019. 10.15171/ijhpm.2018.132 PMC6499905 31050968

[23] United Nations Office for the Coordination of Humanitarian Affairs (UNOCHA). Syrian Arab Republic Humanitarian Response Plan 2018. 2017.

[24] DeJong J, Ghattas H, Bashour H, Mourtada R, Akik C, Reese-Masterson A (2017). Reproductive, maternal, neonatal and child health in conflict: a case study on Syria using countdown indicators. BMJ Glob Health.

[25] Benova L, Cumming O, Campbell OMR (2014). Systematic review and meta-analysis: association between water and sanitation environment and maternal mortality. Tropical Med Int Health.

[26] Campbell OMR, Graham WJ (2006). Strategies for reducing maternal mortality: getting on with what works. Lancet.

[27] Girard AW, Olude O (2012). Nutrition education and Counselling provided during pregnancy: effects on maternal, neonatal and child health outcomes. Paediatr Perinat Epidemiol.

[28] Urdal H, Che CP (2013). War and gender inequalities in health: the impact of armed conflict on fertility and maternal mortality. International Interactions.

[29] Bashour H (2015). Let's not forget the health of the Syrians within their own country. Am J Public Health.

[30] Sweileh WM (2018). Bibliometric analysis of peer-reviewed literature on Syrian refugees and displaced people (2011–2017). Confl Heal.

[31] Ataullahjan A, Gaffey MF, Sami S, Singh NS, Tappis H, Black RE, et al. Investigating the delivery of health and nutrition interventions for women and children in conflict settings: a collection of case studies from the BRANCH Consortium. Confl Heal. 2020;14(1). 10.1186/s13031-020-00276-y. 10.1186/s13031-020-00276-y PMC7254714 32514294

[32] World Health Organization (WHO). WHO vaccine-preventable diseases: monitoring system. 2018 Global Summary 2018. Available from: http://apps.who.int/immunization_monitoring/globalsummary/countries?countrycriteria%5Bcountry%5D%5B%5D=SYR&commit=OK.

[33] World Health Organization (WHO). Syrian Arab Republic - HeRAMS reports. 2018. Available from: http://www.emro.who.int/syr/information-resources/herams-reports.html.

[34] Reuters T. EndNote X7. Philadelphia, PA, USA: Thomson Reuters; 2013.

[35] Harvard Humanitarian Initiative. KoBoToolbox Available from: https://www.kobotoolbox.org/.

[36] Environmental Systems Research Institute. ArcGIS Online. Redlands. 2008; Available from: https://www.arcgis.com/index.html.

[37] Dedoose Version 8.1.8, web application for managing, analyzing, and presenting qualitative and mixed method research data. 2018. www.dedoose.com.

[38] Downe-Wamboldt B (1992). Content analysis: method, applications, and issues. Health care for women international.

[39] Inter-Agency Standing Committee. L3 IASC System-wide response activations and deactivations. 2017. Available from: https://interagencystandingcommittee.org/iasc-transformative-agenda/news-public/l3-iasc-system-wide-response-activations-and-deactivations.

[40] United Nations Office for the Coordination of Humanitarian Affairs. Humanitarian Programme Cycle. Available from: https://www.humanitarianresponse.info/en/programme-cycle/space.

[41] United Nations Office for the Coordination of Humanitarian Affairs (UNOCHA). Humanitarian needs comparison tool. 2014.

[42] de Lima PA, Southgate R, Ahmed H, O’Connor P, Cramond V, Lenglet A. Infectious disease risk and vaccination in northern Syria after 5 years of civil war: the MSF experience. PLoS Currents. 2018;10. 10.1371/currents.dis.bb5f22928e631dff9a80377309381feb PMC5815631 29511602

[43] World Health Organization Regional Office for the Eastern Mediterranean (WHO-EMRO). Implementation of IMCI in Syrian Arab Republic. 2004. Available from: http://www.emro.who.int/child-health/strategy-implementation/implementation-of-imci-in-syrian-arab-republic.html.

[44] Health Cluster - Amman Cross-Border Hub. Health Services and Population Status Report: Southern Syria. 2018.

[45] United Nations Office for the Coordination of Humanitarian Affairs (UNOCHA). Syrian Arab Republic - Administrative Boundaries, Populated Places. 2018. Available from: https://data.humdata.org/dataset/syrian-arab-republic-administrative-boundaries-populated-places.

[46] Medecins Sans Frontieres. Morbidity, healthcare needs and barriers to access medical care amongst local and displaced populations in West Dar’a and Quneitra, Southern Syria. 2018.

[47] Medecins Sans Frontieres. East Dar'a, Syria - Baseline Assessment. 2016.

[48] World Health Organization (WHO). Syrian Arab Republic: WHO and UNICEF estimates of immunization coverage: 2017 revision. 2018.

[49] World Health Organization (WHO), United Nations Children's Fund (UNICEF). Syrian Arab Republic Situation Report No. 1. 2013. Contract No.: March 2018.

[50] Muhjazi G, Bashour H, Abourshaid N, Lahham H (2013). An early warning and response system for Syria. Lancet.

[51] Kristina MC, Susan TC, Andrew TB, Colleen H, Mamunur Rahman M, Peter M, et al. Real-time surveillance in emergencies using the Early Warning Alert and Response Network. Emerging Infectious Disease Journal. 2017;23(13). 10.3201/eid2313.170446 PMC5711309 29155660

[52] Al-Hammami H, Taleb MJ, Alsharif MN (2017). Prevalence of cesarean section at Al Tawlid hospital during the Syrian crisis. Journal of Medical Pharmaceutical and Allied Sciences.

[53] Whole of Syria - Nutrition sector. Report on the Knowledge, Attitude and Practices survey - Infant and Young Child Feeding. 2017.

[54] Medecins Sans Frontieres. East Dar'a, Syria - First Follow-up Assessment. 2017.

[55] Syrian Central Bureau of Statistics, League of Arab States. Family Health Survey in Syrian Arab Republic 2009. 2011.

[56] Ekzayez A (2017). The association between war exposures and health service utilisation in northern Syria: an observational study: London School of Hygiene and Tropical Medicine.

[57] Bashour H, Abdulsalam A (2005). Syrian women's preferences for birth attendant and birth place. Birth..

[58] Huster KMJ, Patterson N, Schilperoord M, Spiegel P (2014). Cesarean sections among Syrian refugees in Lebanon from December 2012/January 2013 to June 2013: probable causes and recommendations. Yale J Biol Med.

[59] Sen K, Al-Faisal W, AlSaleh Y (2012). Syria: effects of conflict and sanctions on public health. J Public Health.

[60] Garry S, Checchi F, Cislaghi B (2018). What influenced provision of non-communicable disease healthcare in the Syrian conflict, from policy to implementation? A qualitative study. Confl Heal.

[61] van der Wal R. Humanitarian intervention in a changing world: Need for a new model of care. Humanitaire [En ligne]. 2015;41.

[62] Donini A, Maxwell D (2013). From face-to-face to face-to-screen: remote management, effectiveness and accountability of humanitarian action in insecure environments. International Review of the Red Cross.

