# Supplementary material for: Responding to health needs of women, children and adolescents within Syria during conflict: intervention coverage, challenges and adaptations
Source: Confl Health. 2020 May 29;14:37. doi: 10.1186/s13031-020-00263-3 (PMC7278078; doi:10.1186/s13031-020-00263-3)
Supplement: Supplementary file 1 — Additional file 1. Desk review findings. [file 13031_2020_263_MOESM1_ESM.docx]

**Table S1 – Descriptive and quality indicators of included publications**

| **Publication** | **Publication type** | **Design** | **Survey type** | **Geographic area covered** | **Target population** | **Clear inclusion/exclusion criteria** | **Sampling strategy** | **Sample size** |
| --- | --- | --- | --- | --- | --- | --- | --- | --- |
| **Central Bureau of Statistics (2006)^1^** | Grey | Cross-sectional | Population-based | National | Residents (*pre-conflict*) | Yes | Two-stage stratified cluster sampling (PPS) | 11,017 |
| **Central Bureau of Statistics (2009)^2^** | Grey | Cross-sectional | Population-based | National | Residents (*pre-conflict*) | Yes | Two-stage stratified random sampling | 17,565 |
| **ACAPS (2014)^3^** | Grey | Cross-sectional | Facility based | Aleppo University Hospital, Aleppo | N/A | No | All facility deliveries in 2011 and 2013 | N/A |
| **Central Bureau of Statistics-UNICEF (2014)^4^** | Grey | Cross-sectional | Population-based | National (*excluding Ar-Raqqa governorate*) | IDPs | Yes | Simple random cluster sampling (PPS) | 3,514 |
| **PAC (2014)^5^** | Grey | Cross-sectional | Population-based | Idleb | Host and IDPs | Yes | Two-stage random cluster sampling | 606 |
| **UNESCWA (2014)^6^** | Grey | Estimation | N/A | N/A | N/A | No | N/A | N/A |
| **UN (2015)^7^** | Grey | Estimation | N/A | N/A | N/A | No | N/A | N/A |
| **MSF (2016)^8^** | Grey | Cross-sectional | Population-based | East Dar'a | Host and IDPs | Yes | Multi-stage cluster sampling and systematic random HH sampling | 764 |
| **Al-Hammami et al (2017)^9^** | Peer-reviewed | Cross-sectional | Facility based | Al-Tawlid University Hospital, Damascus | N/A | No | All facility deliveries from 2010 until mid-2017 | 90,054 |
| **MSF (2017)^10^** | Grey | Cross-sectional | Population-based | East Dar'a | Host and IDPs | Yes | Multi-stage cluster sampling and systematic random HH sampling | 959 |
| **PAC (2017)^11^** | Grey | Cross-sectional | Population-based | Idleb | Host and IDPs | Yes | Two-stage cluster sampling (PPS) and systematic random HH sampling | 578 |
| **PAC (2017)^12^** | Grey | Cross-sectional | Population-based | Eastern Ghouta (*only villages considered secure and accessible*) | N/A | Yes | Two-stage cluster sampling (PPS) and simple random sampling in rural clusters, and systematic random sampling in urban clusters | 314 |
| **WoS Nutrition sector (2017)^13^** | Grey | Cross-sectional | Population-based | Aleppo and Idlib (*excluding 937 inaccessible communities and 316 camps*) | Host and IDPs | Yes | Two-stage cluster sampling (PPS) | 976 |
| **Syrian Development International (2017)^14^** | Grey | Cross-sectional | Population-based | Eastern Ghouta (*five accessible sub-districts: Kafr Batna, Arbin, Duma, Harasta, Nashabiyeh*) | N/A | Not reported | Cluster sampling (PPS) and simple random HH sampling | 627 |
| **Union of Medical Care and Relief Organizations (2017)^15^** | Grey | Cross-sectional | Population-based | Al Lajat area, Dar’a (*two districts: Izra and As-sanamayn*) | Host and IDPs | Yes | Cluster sampling (PPS); simple or systematic random HH sampling | 663 |
| **World Vision (2017)^16^** | Grey | Cross-sectional | Facility based | Idleb, Mariam Hospital | N/A | No | All facility deliveries in July 2016 | 160 |
| **Aburas et al (2018)^17^** | Peer-reviewed | Cross-sectional | Facility based | The Brotherhood Medical Center, Atimah village, Idleb | N/A | No | All facility deliveries in September 2017 | 231 |
| **Al-Ameen Organization (2018)^18^** | Grey | Cross-sectional | Population-based | Al-Bab district, Aleppo (*three sub-districts: Ar-Ra'ee, Al Bab and Arima*) | Host and IDPs | Yes | Two-stage cluster sampling (PPS); simple or systematic random HH sampling | 480 |
| **de Lima et al (2018)^19^** | Peer-reviewed | Cross-sectional | Population-based | Kobane city | N/A | Yes | Simple random HH sampling | 212 |
| **MSF (2018)^20^** | Grey | Cross-sectional | Population-based | West Dar’a and Quneitra  (*excluding ISIS and government controlled areas, and camps*) | Host and IDPs | Yes | Two-stage cluster random sampling | 754 |
| **WHO-UNICEF (2018)^21^** | Grey | Estimation | N/A | National | N/A | No | N/A | N/A |

**Abbreviations**: IDP = Internally Displaced Persons; PPS = Probability Proportional to Size; HH = Households

**Table S2 – Reproductive and Maternal Health indicators in Syria over time before and during conflict (Women of reproductive age 15-49 years)**

| **Year**  **Indicator** | **Before conflict** | **2011** | **2012** | **2013** | **2014** | **2015** | **2016** | **2017** |
| --- | --- | --- | --- | --- | --- | --- | --- | --- |
| Contraceptive prevalence rate (%) | 53.9^2^ |  |  | 47§^6^ | 57.2§^7^ |  | Dar’a: 16.9^8^ | Dar’a: 7.9^10^ |
| Need for family planning satisfied (%) | 83.6^2^ |  |  | 77.3¥^6^ | 78.4¥^7^ |  | Dar’a: 45.1¥^8^ | Dar’a: 35.5¥^10^ |
| ANC 1+ (%) | 87.7^2^ |  |  | 62§^6^ |  | Dar’a: 87.1^8^ | Dar’a: 83.3^10^ | Dar’a/Quneitra: 80.3^20^ |
| ANC 4+ (%) | 63.7^2^ |  |  |  |  |  |  |  |
| Neonatal Tetanus protection (%) | 37.8^1^ |  |  |  |  |  | Dar’a: 19.8‡^8^ | Dar’a: 24‡^10^ |
| Skilled attendant at birth (%) | 96.2^2^ |  |  | 72§^6^ |  | Dar’a:95^8^ | Dar’a:91^10^ | Aleppo/Idleb:97.8^13^ |
| C-section rate (Population) (%) | 26.4^2^ |  |  |  |  | Dar’a:26.3^8^ | Dar’a:24.4^10^ | Aleppo/Idleb:26.6^13^  Dar’a/Quneitra: 23.2^20^ |
| C-section rate (Hospitals) (%) |  | AUH:42.5^3^ |  | AUH: 55.7^3^ |  |  | MH: 51.2^^16^ | BMC: 31.2^17^  3 Hospitals in Dar’a/Quneitra: 41^20^ |
| C-section rate (Dar Al-Tawlid – Damascus) (%) | 29.2^9^ | 32.3^9^ | 33.3^9^ | 43.4^9^ | 46.7^9^ | 42.8^9^ | 46.3^9^ | 50.8*^9^ |
| PNC within 40 days (%) | 27.2^2^ |  |  |  |  |  |  |  |

§Estimate

¥Estimates were calculated using the contraceptive prevalence rate conversion using Countdown Technical Notes^22^

^Data from the month of July only

*January to June 2017

‡At least 1 shot

AUH: Aleppo University Hospital; MH: Mariam Hospital; BMC: The Brotherhood Medical Center.

*N.B.* The year is selected as the midpoint of the recall period in household surveys.

**Table S3 – Child Health indicators in Syria over time before and during conflict**

| **Year**  **Indicator** | **Before conflict** | **2011** | **2012** | **2013** | **2014** | **2015** | **2016** | **2017** |
| --- | --- | --- | --- | --- | --- | --- | --- | --- |
| Measles immunization coverage (12-23 months) (%) | 81.9^2^ | 88* | 70* | 75* | 66* | 64*  Kobani: 8.2^19^ | 69*  Dar’a: 58.1†^8^ | 76*  Dar’a/Quneitra:88.1†^20^ |
| Three doses of combined DTP3 vaccine (12-23 months) (%) | 82.1^2^ | 91* | 64* | 60* | 62* | 60*  Kobani: 12.2^19^ | 61*  Dar’a: 72.9†^8^ | 67*  Dar’a/Quneitra: 43.8†‡^20^ |
| Three doses of combined Hib vaccine (12-23 months) (%) | 99* | 91* | 64* | 60* | 62* | 60* | 61* | 67*  Dar’a/Quneitra: 51.2†‡^20^ |
| One dose of BCG vaccine (0-11 months) (%) | 100* | 100* | 84* | 86* | 83* | 68*  Kobani: 19.6^19^ | 96*  Dar’a: 92.6^8^ | 80*  Dar’a: 88.7§^10^  Dar’a/Quneitra:88.4§^20^ |
| Three doses of Polio vaccine (12-23 months) (%) | 99* | 76* | 63* | 67* | 68*  98¥¤^4^ | 66*  Kobani: 22.5^19^ | 64*  Dar’a: 91.9†^8^ | 69*  Dar’a: 52.4§^10^  Dar’a/Quneitra:97.7†‡^20^ |
| Care seeking for pneumonia (0-59 months) (%) | 85.7^2^ |  |  |  |  |  | Dar’a: 89^8^ |  |
| Antibiotic treatment for pneumonia (0-59 months) (%) | 63.9^2^ |  |  |  |  |  | Dar’a:45.4^₳8^ |  |
| Oral rehydration therapy and continued feeding (0-59 months) (%) | 84.4^2^ |  |  |  |  |  | Dar’a:55.7^$8^ |  |

*Official country estimates^21^

†At least 1 dose

‡Children 13-59 months

§Children 0-59 months

¥Children 6-59 months

¤Among internally displaced persons, excluding Ar-Raqqa governorate

^₳^Among those who sought care at a health facility

^$^Children who received more fluids during the last diarrheal episode

**Table S4 – Nutrition health indicators in Syria over time before and during conflict**

| **Year**  **Indicator** | **Before conflict** | **2011** | **2012** | **2013** | **2014** | **2015** | **2016** | **2017** |
| --- | --- | --- | --- | --- | --- | --- | --- | --- |
| Underweight prevalence (6-59 months) (%) | 10.3§^2^ |  |  |  | 13.8§¤^4^  Idleb: 6.3^5^ |  |  | Idleb: 6.6^11^  East Ghouta: 9.6^14^; 22.4^12^  Al-Bab: 12.4^18^  Al-Lajat: 16.0^15^ |
| Stunting prevalence (6-59 months) (%) | 23§^2^ |  |  |  | 22.3§¤^4^  Idleb: 22.9^5^ |  |  | Idleb: 14.2^11^  East Ghouta: 30.5^14^; 36^12^  Al-Bab: 25.6^18^  Al-Lajat: 27.5^15^ |
| Wasting prevalence (6-59 months) (%) | 9.3§^2^ |  |  |  | 7.2§¤^4^  Idleb: 1.13^5^ |  |  | Idleb: 2.2^11^  East Ghouta: 2.1^14^; 11.9^12^  Al-Bab: 3.1^18^  Al-Lajat: 7.8^15^ |
| Low birth weight among newborns (%) | 10.3^2^ |  |  |  |  |  |  |  |
| Exclusive breastfeeding (0-5 months) (%) | 42.6^2^ |  |  |  | 59.1¤^4^  Idleb: 21.1^5^ |  |  | Aleppo/Idleb: 30.9^13^  Al-Lajat: 32.6^15^ |
| Early initiation of breastfeeding (%) | 45.5^2^ |  |  |  |  |  |  | Aleppo/Idleb: 37.8^13^ |
| Complementary feeding (6-8 months) | 42.9¶^2^ |  |  |  |  |  |  | Aleppo/Idleb: 86.6^13^  Al-Lajat: 58.8^15^ |
| Vitamin A supplementation (at least 1 dose) (%) | 34.8^2^ | 59* | 78* | 64* | 71* | 62* | 61* | 84* |

*Official country estimates^21^

§Children 0-59 months

¶Children 6-23 months

¤Among internally displaced persons, excluding Ar-Raqqa governorate

**References**

1. Syrian Central Bureau of Statistics. The Syrian Arab Republic Multiple Indicator Cluster Survey (MICS). 2006.
2. Syrian Central Bureau of Statistics, League of Arab States. Family Health Survey in Syrian Arab Republic - 2009. 2011.
3. ACAPS. SNAP: Regional Analysis Syria, Part I: Syria - 03 July 2014. 2014; https://theglobalobservatory.org/wp-content/uploads/2014/07/pdfs_syria_update_july_2014.pdf.
4. Syrian Central Bureau of Statistics, UNICEF. Rapid Nutritional Assessment Report. 2014.
5. Physicians Across Continents. SMART Methodology Nutrition Assessment - Idleb Governorate, Syria. 2014.
6. United Nations Economic and Social Commission for Western Asia (UN ESCWA). The Conflict in the Syrian Arab Republic, Implications for the Macro-Economy & MDGs. 2014; https://www.unescwa.org/publications/conflict-syria-macroeconomic-implications-obstacles-achieving.
7. United Nations Department of Economic and Social Affairs - Population Division. Estimates and Projections of Family Planning Indicators. 2015; http://www.un.org/en/development/desa/population/theme/family-planning/cp_model.shtml.
8. Medecins Sans Frontieres. East Dar'a, Syria - Baseline Assessment. 2016.
9. Al-Hammami H, Taleb MJ, Alsharif MN. Prevalence of Cesarean section at Al Tawlid hospital during the Syrian crisis. Journal of Medical Pharmaceutical and Allied Sciences. 2017;2(12):947-953.
10. Medecins Sans Frontieres. East Dar'a, Syria - First Follow-up Assessment. 2017.
11. Physicians Across Continents. SMART survey report - Idleb Governorate, Syria. 2017.
12. Physicians Across Continents. Nutrition SMART Survey Report - Eastern Ghouta, Syria - November 2017. 2017.
13. Whole of Syria - Nutrition sector. Report on the Knowledge, Attitude and Practices survey - Infant and Young Child Feeding. 2017.
14. Syrian Development International. Report of SMART Nutrition Survey Eastern Ghouta – Rural Damascus of (Syria) during (5/1/2017 to 14/1/2017). 2017.
15. Union of Medical Care and Relief Organizations. SMART Survey Report - Nutrition in Al Lajat, Dar’a South Syria. 2017.
16. World Vision. Committed to Children: Syria Crisis Response Annual Review. 2017.
17. Aburas R, Najeeb A, Baageel L, Mackey TK. The Syrian conflict: a case study of the challenges and acute need for medical humanitarian operations for women and children internally displaced persons. BMC Medicine. 2018;16(1):65-70.
18. Al-Ameen Organization. SMART survey Report: Al-Bab district, Aleppo, North Syria. 2018.
19. de Lima Pereira A, Southgate R, Ahmed H, O’Connor P, Cramond V, Lenglet A. Infectious disease risk and vaccination in Northern Syria after 5 years of civil war: The MSF experience. PLoS Currents. 2018;10.
20. Medecins Sans Frontieres. Morbidity, healthcare needs and barriers to access medical care amongst local and displaced populations in West Dar’a and Quneitra, Southern Syria. 2018.
21. World Health Organization (WHO). Syrian Arab Republic: WHO and UNICEF estimates of immunization coverage: 2017 revision. 2018.
22. Bhutta ZA, Chopra M, Axelson H, et al. Countdown to 2015 decade report (2000-10): taking stock of maternal, newborn, and child survival. The Lancet. 2010;375(9730):2032-2044.
