# Supplementary material for: Responding to health needs of women, children and adolescents within Syria during conflict: intervention coverage, challenges and adaptations
Source: Confl Health. 2020 May 29;14:37. doi: 10.1186/s13031-020-00263-3 (PMC7278078; doi:10.1186/s13031-020-00263-3)
Supplement: Supplementary file 2 — Additional file 2. Desk review search strategy and inclusion criteria. [file 13031_2020_263_MOESM2_ESM.docx]

## **Searched databases and search strategy**

We searched Medline, PubMed, Scopus, Popline and Index Medicus for WHO Eastern-Mediterranean Region (EMR) for peer-reviewed literature. Medical Subject Heading terms and keyword search strategies were used with various combinations of terms related to relevant RMNCAH&N indicators, with the additional descriptors of Syria. Additional peer-reviewed documents were captured through a Google Scholar search, a purposive search of relevant journals (e.g. Journal of Health Sciences from Damascus University), and a hand search of bibliographies of included articles. The following four databases relevant to the humanitarian response to the Syrian crisis were searched for grey literature documents: Relief Web, UNHCR Syria Regional Refugee Response Portal, United Nations Development Programme and WHO-EMRO websites. Taking into consideration the inconsistencies in indexing grey literature reports in the searched databases, we also performed a thorough Google and bibliography hand search.

Below is the search strategy used for PubMed, which was adapted for the other databases:

## PubMed search strategy:

Search: ((((((((((((((premature birth OR premature births[MeSH Terms]))) OR premature birth[Text Word])) OR (((domestic violence[MeSH Terms]) OR family violence[MeSH Terms]) OR (domestic violence OR family violence[Text Word]))) OR ((child mortality[MeSH Terms]) OR ((((under-five) OR (0-59 months) OR (0-60 months) OR (&lt;5) OR (less than five) OR (less than 5) AND Text Word)) AND mortality[Text Word]))) OR (((birth rate, age specific[MeSH Terms]) OR (((adolescent pregnancy[MeSH Terms]) OR pregnancies, teen[MeSH Terms]) OR adolescence, pregnancy in[MeSH Terms])) OR (((pregnancy in adolescence) OR (adolescen* AND birth rate) OR (teen pregnan* OR (adolescen* AND fertil*) AND Text Word)))) OR ((age specific fertility rate[MeSH Terms]) OR (fertility OR (fertility rate*) AND Text Word))) OR ((stillbirth[MeSH Terms]) OR ((stillbirth*) OR (still-birth*) OR (still birth*))))) OR ((((((((((((((maternal death* OR maternal mortality*[Text Word]))) OR maternal mortality[MeSH Terms])) OR (((care, prenatal[MeSH Terms]) OR care, perinatal[MeSH Terms]) OR (perinatal care OR prenatal care OR antenatal care[Text Word]))) OR (((care, postnatal[MeSH Terms]) OR care, postpartum[MeSH Terms]) OR puerperium[MeSH Terms])) OR (((delivery, obstetric[MeSH Terms]) OR ((emergency care) AND (obstetric*) AND MeSH Terms)) OR ((obstetric deliver*) OR (emergency obstetric care) AND Text Word))) OR ((skilled birth attendan*[Text Word]) OR skilled deliver*[Text Word])) OR (((family planning[MeSH Terms]) OR family planning services[MeSH Terms]) OR (family planning AND (program* OR use* OR usage* OR utilization OR method*)))) OR (((cesarean section[MeSH Terms]) OR abdominal delivery[MeSH Terms]) OR (cesarean section OR abdominal delivery[Text Word]))) OR (((abortion induced[MeSH Terms]) OR miscarriage[MeSH Terms]) OR (abortion OR induced abortion OR miscarriage OR spontaneous abortion OR unwanted abortion OR medical abortion OR termination of pregnancy[Text Word])))) OR ((((reproductive health services[MeSH Terms]) OR reproductive health service*[Text Word])) OR ((((accessibility of health services[MeSH Terms]) OR (access* AND and health service*[Text Word]))) AND (((((women[MeSH Terms]) OR (woman OR women[Text Word])) OR (child* OR infant*[Text Word])) OR child[MeSH Terms]) OR infant[MeSH Terms]))))) OR ((((((((((measles vaccine[MeSH Terms]) OR measles, mumps, rubella vaccine[MeSH Terms]) OR vaccine, measles mumps rubella[MeSH Terms])) OR ((measles[Text Word]) AND vaccin*[Text Word]))) OR ((((tetanus[MeSH Terms]) AND (neonatal* OR newborn*[Text Word]))) OR ((tetanus[MeSH Terms]) AND infant, newborn[MeSH Terms]))) OR ((((((HIB OR hemophilus influenzae meningitis type b) AND vaccin*) OR PRP vaccin*[Text Word]))) OR ((((hib meninigitis [MeSH Terms]) OR hemophilus influenzae meningitis type b[MeSH Terms])) AND ((vaccination[MeSH Terms]) OR mass vaccination[MeSH Terms])))) OR ((((pneumonia[MeSH Terms]) OR respiratory infections[MeSH Terms])) OR ((pneumonia OR lung infect* OR respiratory infect*[Text Word])))) OR (((((((diphtheria tetanus pertussis vaccine[MeSH Terms]) OR diphtheria pertussis tetanus vaccine[MeSH Terms]) OR tetanus toxoid[MeSH Terms])) OR ((((mass vaccination[MeSH Terms]) OR vaccination[MeSH Terms])) AND ((((tetanus[MeSH Terms]) OR diphteria[MeSH Terms]) OR pertussis[MeSH Terms]) OR cough, whooping[MeSH Terms])))) OR (((diphteria OR tetanus OR pertussis OR diphtheria tetanus pertussis OR tetanus toxoid OR diphteria toxoid[Text Word])) AND vaccin*[Text Word])))) OR (((((((((low birth weight[MeSH Terms]) OR birth weight, low[MeSH Terms]) OR low birth weight[Text Word])) OR ((((underweight[MeSH Terms]) OR undernutrition[MeSH Terms]) OR ((wasting disease[MeSH Terms]) OR wasting syndrome[MeSH Terms])) OR ((((infant malnutrition[MeSH Terms]) OR child malnutrition[MeSH Terms]) OR malnutrition, protein energy[MeSH Terms]) OR nutritional status[MeSH Terms]))) OR ((wasting OR underweight OR stunting OR stunted OR undernutrition OR undernourished OR malnutrition OR malnourish* OR acute malnutrition OR chronic malnutrition OR nutritional status[Text Word])))) OR ((((((breastfeeding[MeSH Terms]) OR breastfeeding, exclusive[MeSH Terms]) OR (breastfeeding OR breastfe*[Text Word]))) OR ((complementary feeding[MeSH Terms]) OR weaning[MeSH Terms])) OR ((complementary food* OR complementary feeding OR weaning OR infant food* OR infant feeding OR solid food*[Text Word])))) OR ((((vitamin A[Text Word]) AND supplement*[Text Word])) OR ((vitamin a[MeSH Terms]) AND supplement*[Text Word]))))) AND ((syria[MeSH Terms]) OR syria*[Text Word])

## **Inclusion and exclusion criteria**

We included original research studies and grey literature documents that reported on 1) relevant RMNCAH&N coverage indicators among the Syrian population residing in Syria, as well as 2) delivered interventions and factors affecting their implementation. We only included quarterly, midyear and annual grey literature reports (excluded weekly and monthly reports). Findings relevant to Syrian refugees residing in neighboring countries were excluded from this analysis.
