# Supplementary material for: Responding to health needs of women, children and adolescents within Syria during conflict: intervention coverage, challenges and adaptations
Source: Confl Health. 2020 May 29;14:37. doi: 10.1186/s13031-020-00263-3 (PMC7278078; doi:10.1186/s13031-020-00263-3)
Supplement: Supplementary file 3 — Additional file 3. Key informant interview guide. [file 13031_2020_263_MOESM3_ESM.docx]

**Interview Guide:**

**Decision makers**

I: Thank you for accepting to have this interview today, as you know we are interested in understanding Reproductive, Maternal, Newborn, Child, Adolescent Health & Nutrition intervention delivery in conflict. We know conflict is a particularly difficult context to work in, and our effort is focused on understanding how you deliver health services in these difficult contexts. We are speaking to a range of individuals involved in health provision during conflict including different levels of NGO staff, UN officials, and government officials. In Syria, we are focusing on the period between 2013 till present. I don’t want to forget anything we discuss today, so would you mind if I record the interview? This recording is for our records and will only be shared with the research team.

**if agrees put on recorder**

**if does not agree**

I: That’s okay I can take written notes instead, do let me know if you change your mind at any point.

I: Before we begin, I will read the informed consent form for you, and I will highlight a few items. First, ensuring your privacy is important to us, and we will remove all personal identifiers from our data. All recordings and notes will be assigned a code so that they are not linked to your name. The key to the codes will be kept in a password protected file which only the research staff can access. We will not link your name, or your position at your organization with your data in any of our documentation. Second, if at any time during the interview you feel uncomfortable and would like to halt the interview please let me know. Feel free to ask me any questions. When you feel comfortable doing so, please let me know so that I note on the form your decision to participate or not in this study.

**Once participant has provided oral consent**

I: Thank you, do you have any questions before we begin?

**1.** **Can you explain to me the role of your organization with regards to providing Reproductive, Maternal, Newborn, Child, Adolescent, Health and Nutrition interventions/programs?**

1. Policy development
2. Funding/financing
3. Technical assistance
4. Direct provision of clinical services
5. Provision of non-clinical services

**2. What activities [services or programs] does your organization currently deliver?**

1. Adolescence
2. Pre-pregnancy
3. Pregnancy (antenatal)
4. Childbirth
5. Postnatal (mother)
6. Postnatal (newborn)
7. Infancy and childhood

Since the start of the conflict, have there been any change(s) in the types of interventions delivered? If yes, what are these? When did the change(s) take place? Why did the change(s) take place?

Breastfeeding and IYCF probes, please inquire if there were any:

1. Interventions supporting milk expression?
2. Facilities where mothers can store milk?
3. Interventions supporting non-breastfed children? E.g. Wet-nursing or informal milk sharing?
4. Interventions to support complementary feeding of children from 6 to 24 months?
5. Interventions to support micronutrient supplementation of children from 6 to 24 months?

**2.1. At what level are decisions made for the activities to be delivered?**

**2.2 Can you explain what considerations factored into the decision to not provide [ ] service?**

**3. *If the interviewee is a decision-maker*, what source(s) of evidence informed and/or continues to inform your decisions on which interventions/programs to deliver? And how?**

1. Formal or informal needs assessments or surveys
2. Surveillance data
3. Scientific/academic literature
4. National or international guidelines
5. Cost-effectiveness studies
6. Other? Specify

**4. What factors have influenced in setting/selecting the Reproductive, Maternal, Newborn, Child, Adolescent Health and Nutrition intervention/programs priorities?**

1. Availability of certain cadres of health workers (retention of workforce, recruitment of new personnel, training process, etc.)
2. The availability of certain commodities (stockpiles, commodity donations, resulting in adjustment in interventions/programs, etc.)
3. The level of funding received (primary source, source-related restrictions, financial constraints, ability to liquidate funds, capital, need to contact head offices, etc.)
4. Any other elements or resources? Specify (difficulties in procurement, availability, etc.)
5. International agenda
6. Organization’s expertise in one area of health

Have these factors changed over time?

**5. Can you describe how you negotiate competing priorities within your organization? Can you give me an example?**

In what sense are priorities competing? (i.e. time, workforce, resources, capital etc.)

**6. Were there any changes to the services that were planned compared to those that were delivered?**

- 1. Can you describe how these services changed?
  2. What factors influenced changes?

**7. To what extent does your organization interact with government entities, UN agencies, NGOs (whether local or international)? (e.g. attend cluster meetings, coordination of service delivery, funding, etc.) Have these interactions changed over time?**

7.1. Does your organization subcontract services to other organizations?

- - What types of services are subcontracted?
  - Where are services subcontracted?
  - Can you explain why services are subcontracted? Can you give me an example?
  - Can you describe to what extent the subcontracted organization can influence the services or types of services delivered?

**8. How do security constraints impact the implementation of RMNCAH&N interventions/programs? Have these changed over time?**

8.1. Can you describe how you manage security concerns to ensure implementation of RMNCAH&N interventions/ programs?

**9. Who is your beneficiary population? What influences who you deliver your services to?**

- 1. Were there any subpopulations that were particularly hard to access? Can you describe this group? Were there any special efforts to try to access these subpopulations? Can you give an example?
  2. Did you deliver the same services differently to the different subpopulations in the same region? Can you explain what is underlying this difference?
  3. Did the sociocultural context affect one health domain in particular? Can you give me an example?

**10. Can you describe any other factors that affected the delivery or implementation of interventions**/**programs?**

**11. Were any formal or informal evaluations conducted to assess the effectiveness of delivery and/or implementation of Reproductive, Maternal, Newborn, Child, Adolescent Health and Nutrition interventions/programs? Can you share it with us?**

**Interview Guide:**

**Representatives of implementing organizations**

I: Thank you for accepting to have this interview today, as you know we are interested in understanding Reproductive, Maternal, Newborn, Child, Adolescent Health & Nutrition intervention delivery in conflict. We know conflict is a particularly difficult context to work in, and our effort is focused on understanding how you deliver health services in these difficult contexts. We are speaking to a range of individuals involved in health provision during conflict including different levels of NGO staff, UN officials, and government officials. In Syria, we are focusing on the period between 2013 till present. I don’t want to forget anything we discuss today, so would you mind if I record the interview? This recording is for our records and will only be shared with the research team.

**if agrees put on recorder**

**if does not agree**

I: That’s okay I can take written notes instead, do let me know if you change your mind at any point.

I: Before we begin, I will read the informed consent form for you, and I will highlight a few items. First, ensuring your privacy is important to us, and we will remove all personal identifiers from our data. All recordings and notes will be assigned a code so that they are not linked to your name. The key to the codes will be kept in a password protected file which only the research staff can access. We will not link your name, or your position at your organization with your data in any of our documentation. Second, if at any time during the interview you feel uncomfortable and would like to halt the interview please let me know. Feel free to ask me any questions. When you feel comfortable doing so, please let me know so that I note on the form your decision to participate or not in this study.

**Once participant has provided oral consent**

I: Thank you, do you have any questions before we begin?

1. **Can you explain to me what your role as [position] with regards to providing Reproductive, Maternal, Newborn, Child, Adolescent Health and Nutrition interventions/programs entails?**

- 1. Can you describe your educational background?

1. **What activities [services or programs] does your organization currently deliver?**
   1. Adolescence
   2. Pre-pregnancy
   3. Pregnancy (antenatal)
   4. Childbirth
   5. Postnatal (mother)
   6. Postnatal (newborn)
   7. Infancy and childhood

Since the start of the conflict, have there been any change(s) in the types of interventions delivered? If yes, what are these? When did the change(s) take place? Why did the change(s) take place?

Breastfeeding and IYCF probes, please inquire if there were any:

1. Interventions supporting milk expression?
2. Facilities where mothers can store milk?
3. Interventions supporting non-breastfed children? E.g. Wet-nursing or informal milk sharing?
4. Interventions to support complementary feeding of children from 6 to 24 months?
5. Interventions to support micronutrient supplementation of children from 6 to 24 months?

**2.1 Can you explain what considerations factored into the decision not to provide [ ] service?**

**3.1 Can you describe obstacles you faced delivering this intervention/program? Can you give me examples?**

- Did any of the obstacles you faced require revisiting the planned delivery method?

**3.2 Can you describe any innovative approaches that were used to overcome these obstacles?**

- Were any other modes of delivery considered but eliminated as not feasible? Can you give me examples?

**3.3 Can you describe where your recipients access your interventions/programs?**

- Can you describe the considerations that led to that location being identified as the place of delivery?
- How did the use of this location assist in the delivery of the intervention/program?
- How did the use of this location act as barrier to the delivery of the intervention/program?

**3.4 Were multiple interventions/programs packaged together?**

**3.5 Who accessed your interventions/programs? Can you describe this group? (Gender? Age? Educational status? Refugees and/or IDPS? Camp residents and/or dispersed population? Etc.) What influences who you deliver your services to?**

- Who were the intended recipients or beneficiaries of interventions/programs?
- Were there any regional variations in the type of recipient who accessed interventions/programs?

Were there differences between intended recipients and actual recipients? If so, why do you think that is?

- Did you deliver the same services differently to different subpopulations in the same region? Can you explain what is underlying this difference?
- Were there any groups that this delivery method did not work well for? Can you describe this group?
- Were there any special efforts to try to access this group? Can you give an example?
- Did the sociocultural context affect service provision in one health domain in particular? Can you give me an example?

1. **How do security constraints impact the implementation of Reproductive, Maternal, Newborn, Child, Adolescent Health and Nutrition interventions/programs?**
   1. Can you describe how your team managed security concerns to ensure implementation of Reproductive, Maternal, Newborn, Child, Adolescent Health and Nutrition interventions/ programs?
2. **Can you describe any other factors that affected the delivery or implementation of interventions?**
3. **Were there any changes to the services that were planned compared to those that were delivered?**

**6**.1 Can you describe how these services changed?

**6**.2 What factors influenced changes?

**7. Were any formal or informal evaluations conducted to assess the effectiveness of delivery and/or implementation of Reproductive, Maternal, Newborn, Child, Adolescent Health and Nutrition interventions/programs? Can you share it with us?**
