# Supplementary material for: Responding to health needs of women, children and adolescents within Syria during conflict: intervention coverage, challenges and adaptations
Source: Confl Health. 2020 May 29;14:37. doi: 10.1186/s13031-020-00263-3 (PMC7278078; doi:10.1186/s13031-020-00263-3)
Supplement: Supplementary file 4 — Additional file 4. List of RMNCAH&N indicators used to build Map 1. [file 13031_2020_263_MOESM4_ESM.docx]

**List of indicators used for Map 1**

1. Reproductive and maternal health indicators

- Contraceptive prevalence rate
- Antenatal care (at least one visit)
- Neonatal Tetanus protection
- Skilled attendant at birth
- Proportion of institutional deliveries
- Caesarian section rate (Facility-level)
- Caesarian section rate (Population-level)

1. Child health indicators

- Polio vaccination coverage
- Measles vaccination coverage
- DTP vaccination coverage
- BCG vaccination coverage
- HepB vaccination coverage
- HiB vaccination coverage
- Care seeking for pneumonia
- Diarrhea treatment

1. Nutrition indicators

- Early initiation of breastfeeding
- Exclusive breastfeeding at 6 months
- Continued Breastfeeding at 12-24 months
- Complementary feeding
- Underweight prevalence (children 6-59 months)
- Stunting prevalence (children 6-59 months)
- Wasting prevalence (children 6-59 months)
- Management of severe acute malnutrition (facility-level)
- Minimum diet diversity (children <24 months)
